# Ferulic Acid Combines with Ascorbic Acid to Target MMP9 to Attenuate Cisplatin-Induced Ototoxicity Through the p38MAPK Signaling Pathway

**DOI:** 10.3390/antiox14060619

**Published:** 2025-05-22

**Authors:** Guojun Yang, Na Hu, Jie Gao, Xinzhi Li, Bin Zhang, Ketao Ma

**Affiliations:** 1 Department of Physiology, School of Basic Medicine, Tongji Medical College, Huazhong University of Science and Technology, Wuhan 430030, China; d202081419@hust.edu.cn (G.Y.);; 2 Department of Otolaryngology, The First Affiliated Hospital of Shihezi University, Shihezi 832000, China; 3 The Key Laboratory of Xinjiang Endemic and Ethnic Diseases, Ministry of Education, Shihezi University School of Medicine, Shihezi 832002, Chinalixinzhi@shzu.edu.cn (X.L.); 4 NHC Key Laboratory of Prevention and Treatment of Central Asia High Incidence Diseases, Shihezi 832002, China; 5 Department of Physiology, Shihezi University School of Medicine, Shihezi 832002, China; 6 The Institute for Brain Research, Collaborative Innovation Center for Brain Science, Huazhong University of Science and Technology, Wuhan 430030, China; 7 Hubei Key Laboratory of Drug Target Research and Pharmacodynamic Evaluation, Huazhong University of Science and Technology, Wuhan 430030, China; 8 Department of Pathophysiology, Shihezi University School of Medicine, Shihezi 832002, China

**Keywords:** ferulic acid, ascorbic acid, cisplatin, ototoxicity, apoptosis

## Abstract

Cisplatin (Cis) is a commonly used chemotherapeutic agent for the clinical management of malignant tumors, but its toxic side effects could cause hearing loss, and there is an urgent need to find drugs that ameliorate Cis ototoxicity. Previous studies have found that ferulic acid (FA), a phenolic compound derived from natural plants, exerts antioxidant and anti-inflammatory effects by scavenging free radicals, preventing lipid peroxidation and cell death. Combination therapy, the use of multiple drugs to improve clinical outcomes, has multiple advantages compared to monotherapy. Another small-molecule ascorbic acid (AA) shows robust antioxidant function. However, the optimal route of administration, dosage, concentration, and effective time must be determined. More importantly, whether the combination of FA and AA can improve Cis ototoxicity and reduce the risk of large doses of AA is unclear. This study aims to evaluate the therapeutic potential of FA combined with AA in Cis-induced hearing impairment. In vitro and in vivo experiments were performed to observe the effects of FA, AA, and FA+AA on Cis-induced apoptosis. Compared with the Cis-only group, FA combined with AA ameliorated the Cis-induced decrease in cell viability, production of reactive oxygen species (ROS), and apoptosis of cells to varying degrees, respectively, and the improvement in cell viability, ROS, and apoptosis was even more pronounced with the combination of the two treatments. Network pharmacology combined with transcriptomics and molecular docking results showed that FA and AA could inhibit the Cis-induced apoptosis of cochlear hair cells through Matrix Metalloproteinase 9(MMP9)via the p38 Mitogen-Activated Protein Kinase (p38 MAPK) signaling pathway. In this study, we discovered that FA+AA reduced Cis ototoxicity by suppressing MMP9 in the MAPK signaling pathway.

## 1. Introduction

Cis is a platinum-based chemotherapeutic agent widely used to treat various cancers. However, its clinical utility is limited by severe side effects, particularly ototoxicity and nephrotoxicity, which often necessitate dose reduction or treatment discontinuation. Nearly 1 million people annually are exposed to antineoplastic drugs, with chemotherapy-induced hearing loss estimated to affect 500,000 cases per year [1]. The mechanisms underlying Cis-induced ototoxicity remain incompletely understood but involve DNA damage, oxidative stress, and cochlear cell apoptosis. Cisplatin forms interstrand crosslinks in DNA, triggering (ROS) overproduction, lipid peroxidation, antioxidant depletion, calcium influx, and, ultimately, apoptosis of cochlear hair cells, particularly outer hair cells [2,3,4]. A key contributor to Cis-induced hearing loss is oxidative stress, which damages cochlear structures and activates inflammatory pathways [5,6]. These pathways further amplify inflammation and apoptosis via caspase-8 activation [7,8]. Given these mechanisms, antioxidants such as glutathione, N-acetylcysteine (NAC), and sodium thiosulfate have been explored as protective agents with varying success [9,10,11].

MMP9 a zinc-dependent endopeptidase, plays a crucial role in extracellular matrix (ECM) remodeling and cellular processes such as inflammation, apoptosis, and oxidative stress responses [12]. Elevated MMP9 exacerbates tissue damage by degrading ECM components, rendering cells more susceptible to oxidative injury [13,14,15]. In the auditory system, MMP9 is expressed in the organ of Corti, pillar cells, and spiral ganglion neurons, suggesting a potential role in ototoxicity [16,17,18,19]. MMP9 may serve as a predictor of cochlear implant outcome in the treatment of prelingual deafness [20]. In the model of cochlear blood–labyrinth barrier injury caused by cisplatin, the expression of MMP9 was increased in peristriatal cells. However, the role of MMP9 in Cis-induced damage in cochlear hair cells remains unclear [21]. Notably, MMP9 is regulated by the MAPK pathway, particularly p38 MAPK, which is activated by oxidative stress and inflammation [22,23]. The inhibition of MMP9 has been shown to improve recovery in neural injury models, raising the possibility that targeting MMP9/MAPK signaling could mitigate Cis-induced ototoxicity [22].

Given the limitations of current protective strategies, natural antioxidants like FA and AA have emerged as promising candidates. FA attenuates Cis-induced cytotoxicity by scavenging ROS and boosting endogenous antioxidants [24], while AA has been shown to reduce ototoxicity in models of drug-induced hearing loss [25]. In other models, FA and AA exert a synergistic effect on each other, for instance, isoproterenol-induced myocardial infarction in rats, cardiac toxicity caused by β-adrenergic catecholamines in rats, and skin aging caused by light irradiation [26,27,28]. Since MAPK/MMP9 signaling is a critical mediator of Cis-induced damage, we hypothesize that FA and AA may protect against ototoxicity by modulating this pathway.

Although FA and AA have been individually investigated in Cis-induced ototoxicity, their combined effects on MMP9 through p38 MAPK pathway modulation have not been systematically examined. This study investigates whether FA and AA exert protective effects by suppressing oxidative stress and MMP9 activation via the MAPK pathway, offering potential therapeutic strategies to prevent Cis-associated hearing loss.

## 2. Materials and Methods

### 2.1. Materials

Cis (purity ≥ 99.0%), FA (mixture of isomers, analytical standard), and AA (ACS reagent, ≥99%) were purchased from Sigma-Aldrich (St. Louis, MO, USA). Primary antibodies against Bax (ab32503), Bcl-2 (ab32124), Caspase-3 (ab32351), MYO7A (ab150386), Caspase-9 (ab202068), and Cytochrome C (cyt-C) (ab133504) were purchased from Abcam (Cambridge, UK). Anti-p38 (GB154685-100), anti-p-p38 (GB153380-100), and anti-MMP9 (GB11132-100) were purchased from Sevier Biotechnology Co., Ltd. (Wuhan, China). FITC-Phalloidin (ab176753) and TRITC-Phalloidin (ab176757) were purchased from Abcam (Cambridge, UK). Dulbecco’s modified Eagle’s medium (DMEM), fetal bovine serum (FBS), and penicillin/streptomycin were purchased from Gibco (Grand Island, NY, USA). An apoptosis detection kit was purchased from Vazyme Biotech Co., Ltd. (Nanjing, China). Trypsin was purchased from HyClone Corporation (Logan, UT, USA). The Annexin V-FITC/PI apoptosis kit was manufactured by MultiSciences (Lianke) Biotechnology Co., Ltd. (Hangzhou, China). CellEvent Caspase 3/7 Green Detection Reagent was obtained from Thermo Fisher Scientific (C10423) (Waltham, MA, USA). MitoSOX Red was obtained from Thermo Fisher Scientific (M36008). Dimethyl sulfoxide (DMSO), RIPA buffer, and phenylmethylsulfonyl fluoride (PMSF) were purchased from Solarbio Science & Technology Co., Ltd. (Beijing, China). Paraformaldehyde (4%) and enhanced chemiluminescence detection (ECL) reagents were purchased from Biosharp (Hefei, China). A One-step TUNEL Apoptosis Detection Kit (C1088) was purchased from Biyuntian Biotechnology Co., Ltd. (Shanghai, China). p38 MAPK-IN-1 (HY-12839) was purchased from MedChemExpress (Monmouth Junction, NJ, USA). Anti-β-actin and Anti-GAPDH were purchased from Zhong Shan-Golden Bridge Biological Technology Co., Ltd. (Beijing, China). Annexin V-FITC/PI Apoptosis Kit was purchased from Multisciences (Lianke) Biotech Co., Ltd. (Hangzhou, China). The No. 8 cell clamp was purchased from Guangdong Sude Technology Co., Ltd. (Dongguan, China).

### 2.2. Animal Experiments

All the animal experiments and procedures were approved by the Institutional Animal Care and Use Committee of Shihezi University and complied with the Animal Experiment Ethics Regulations of Shihezi University. Eight-week-old C57BL/6J mice were purchased from Skobes Biotechnology Co., Ltd. (Anyang, China). To investigate the protective effects of FA and AA in vivo, 1 mL of preheated sterile saline was intraperitoneally injected one day before the administration of Cis and/or FA and AA. The mice were intraperitoneally injected with FA at a dose of 100 mg/kg, and the mice were intraperitoneally injected with AA at a dose of 150 mg/kg. Two hours later, 30 mg/kg Cis was intraperitoneally injected once. After Cis administration, mice in all the groups (the Con group, Cis group, FA + Cis group, AA + Cis group, and FA + AA + Cis group) were intraperitoneally injected with 1 mL of prewarmed saline twice a day for seven consecutive days to alleviate nephrotoxicity and dehydration, and then the mice were subjected to auditory brainstem response (ABR) tests. The dose of Cis and the procedure that was used were chosen based on a previous study of mice with hearing loss [29].

### 2.3. Cell Culture

HEI-OC1 cells were kindly donated by Professor Renjie Chai, School of Life Sciences, Southeast University, Nanjing, China. HEI-OC1 cells were cultured in Dulbecco’s modified Eagle’s medium (DMEM) supplemented with 10% fetal bovine serum (FBS) at 33 °C and 5% CO_2_ in a humidified atmosphere. The cells were allowed to reach approximately 80% confluence before the following experiments were performed. The target concentration of Cis was determined and administered to HEI-OC1 cells to induce cell apoptosis; FA (100 μM or 200 μM) and AA (125 μM or 250 μM) were administered to HEI-OC1 cells with Cis. Five groups were included in this study: the HEI-OC1, HEI-OC1 + Cis, HEI-OC1 + FA + Cis, HEI-OC1 + AA + Cis, and HEI-OC1 + FA + AA + Cis groups. The HEI-OC1-alone group was set as the normal control.

### 2.4. Cochlear Explants Culture In Vitro

The cochlea of newly born C57BL/6 mice (3–4 days old) was placed in pre-cooled PBS after removal under a stereomicroscope. The cochlea shell, which had not been fully calcified, was gently peeled off with No. 8 forceps and laid face up on a cell-tak-coated coverslip in the culture medium of DMEM/F12 containing N2/B27 (Thermo Fisher Scientific, Waltham, MA, USA) and ampicillin. The cultures were incubated overnight at 37 °C in a humidified environment containing 5% CO_2_.

### 2.5. Cell Viability Assay

Cell viability was assessed using a CCK-8 assay. HEI-OC1 cells were seeded in a 96-well plate at an optimal density (5000–6000 cells per well) in 100 µL of culture medium and allowed to adhere and grow for 24 h under standard culture conditions (33 °C, 5% CO_2_). Serial dilutions of the test compounds (FA and AA) were prepared in the culture medium and the plates were incubated for the desired treatment duration (24 h) under standard culture conditions. Each group’s HEI-OC1 cells were washed with prewarmed Phosphate-Buffered Saline (PBS) before adding CCK-8 solution and incubating for 2 h at 33 °C in a CO_2_ incubator. The absorbance was measured at 450 nm using a zymograph (Bio-Rad Laboratories, Hercules, CA, USA). Each group was tested three times.

### 2.6. Apoptosis Analysis

TUNEL apoptosis detection kit (Multi Sciences, Hangzhou, China) was used. Briefly, cells were washed once with prewarmed PBS, fixed in 4% paraformaldehyde for 30 min, and then washed again. The cells were incubated with 0.3% Triton X-100 for 5 min at room temperature. In each sample, 5 μL of TDTase is added to 45 μL of Fluorescent Labeling Solution and incubated in the dark at 37 °C for 60 min. The cells were washed three times with PBS and observed under a fluorescence microscope. According to the instructions of the Annexin V-FITC/PI Apoptosis Kit, 5 μL of Annexin V-FITC and 10 μL of PI were added to samples in each group; the stained cells were detected by flow cytometry (BD FACS Aria III, San Jose, CA, USA), and the apoptosis rate was analyzed by FlowJo V10 software.

### 2.7. ROS Assay

DCFH-DA and MitoSox Red (Life Technologies, Invitrogen, Waltham, MA, USA) were employed to determine intracellular and mitochondrial ROS levels, respectively. To visualize ROS generation, HEI-OC1 cells were seeded in 12-well plates at 15,000 cells per well and treated as indicated. Following drug intervention, cells were doubly stained with 20 μmol/L DCFH-DA and 5 μmol/L MitoSox-Red. After 30 min in the dark, the cells were stained with Hoechst 33342 for 10 min to reveal the nuclei. The cells were washed three times with PBS and examined using a Zeiss fluorescence microscope (Jena, Germany).

### 2.8. RNA-Seq and Quantitative PCR

RNA-seq samples were divided into two groups (n = 3), the model group (Cis 30 μM) and the treatment group (FA 200 μM, AA 250 μM, Cis 30 μM). RNA was extracted using the RNeasy Mini Kit (Qiagen, Valencia, CA, USA) according to the manufacturer’s instructions. A NanoDrop 2000 spectrophotometer (Thermo Fisher Scientific) was used to determine RNA purity and quantity. The assessment of RNA integrity was performed using an Agilent 2100 Bioanalyzer (Agilent Technologies, Santa Clara, CA, USA). A TruSeq Stranded mRNA LT Sample Prep Kit (Illumina, San Diego, CA, USA) was used for library construction according to the manufacturer’s instructions. The Illumina HiSeq X Ten platform was used for library sequencing and for generating 150-bp paired-end reads. Raw reads (FASTQ format) were first processed with Trimmomatic, and low-quality reads were removed to obtain clean reads. Differential expression analysis was performed using the DESeq2 (2012) R package. *p* < 0.05, fold change > 2 and FDR < 0.01 indicated significant differential expression. The raw sequencing data were deposited in the NCBI Sequence Read Archive under the following accession numbers: the Cis group: SRR32423921, SRR32423922, SRR32423923; the FA+AA+Cis group: SRR32423918, SRR32423919, SRR32423920.

### 2.9. Western Blotting

After treatment, the cells were washed in cold PBS and lysed with RIPA buffer containing protease inhibitors and phosphatase inhibitors on ice for 20–30 min. The protein concentrations were determined using a BCA kit (Thermo Fisher Scientific, Waltham, MA, USA). Equal amounts of proteins were separated on a Sodium Dodecyl Sulfate Polyacrylamide Gel Electrophoresis and transferred to Polyvinylidene Fluoride (PVDF) membranes. The membranes were then incubated with specific primary antibodies (overnight at 4 °C). Primary antibodies targeting the following proteins were used in this study: β-actin (1:1000), GAPDH (1:1000), Bcl-2 (1:1000), caspase3 (1:1000), cleaved-caspase3 (1:1000), caspase9 (1:1000), cleaved-caspase9 (1:1000), Bax (1:1000), cyt-C (1:1000), MMP9 (1:1000), p38 (1:1000), and p-p38 (1:1000). The next day, the PVDF membranes were incubated with a horseradish peroxidase-labeled secondary antibody (1:10,000) at room temperature for 2 h. After being washed with TBST, the PVDF membranes were incubated with a chemiluminescent solution from the ECL kit and scanned with a chemiluminescent imager. The images were saved for quantitative analysis.

### 2.10. Immunofluorescence

Fixed cochleae and cells were permeabilized (0.5% Triton X-100 in PBS; 30 min), blocked (0.5% BSA in PBST; 1 h), and incubated with primary antibodies against MMP9 (1:250) and p-p38 (1:250) overnight at 4 °C. Then, the samples were treated with a secondary antibody fluorescent antibody in the dark for 2 h. 4′,6-Diamidino-2-phenylindole (DAPI; Sigma Aldrich) was employed to stain for 5 min. For phalloidin staining, the fixed cochlear specimens were treated with phalloidin-iFuor 594 Reagent (Abcam) (1:1000) or phalloidin-iFuor 488 Reagent (Abcam) (1:1000) for 120 min in the dark and subjected to DAPI counterstaining. Differences in staining were examined under a microscope. CellEvent Caspase 3/7 Green Detection Reagent (Thermo Fisher Scientific, C10423) was used. The stock solution was diluted 1:400 with growth medium, and the cells were stained for 30–60 min. The cell nuclei were stained with Hoechst 33342 for 5–10 min in the dark. The cells were washed with PBS thrice and evaluated with a fluorescence microscope. A One-step TUNEL Cell Apoptosis Detection Kit (Beyotime, Shanghai, China, C1088) was used. The cells were fixed with 4% paraformaldehyde for 30 min, washed once with PBS containing 0.3% Triton X-100 PBS, and incubated at room temperature for 5 min. TUNEL solution was added to the sample and incubated at 37 °C for 60 min in the dark. The cells were washed with PBS thrice. The cell nuclei were stained with DAPI for 5 min in the dark. The cells were washed with PBS thrice and evaluated with a fluorescence microscope.

### 2.11. ABR Measurements

Each group of anesthetized mice (100 mg/kg ketamine and 25 mg/kg thiazide sodium, i.p.) conducted ABR analysis to test hearing thresholds before and after drug delivery. Hearing thresholds at four frequencies (8, 16, 24, and 32 kHz) were measured using a TDT system (3 Technology, Gainesville, FL, USA). Three electrodes (stimulating, reference, and ground) were usually placed subcutaneously at the cranial apex, ipsilateral mastoid, and nasal root. The parameters for the elicited responses and acquired signal were as follows: Tone burst length is 5 ms, rise-fall time is 0.5 ms, stimulus frequency is 21.37/s, stacking fold (superposition times) is 500–1000, magnification is 20, bandpass is 0.3–3 kHz, sound intensity fluctuation is 5 dB, and SPL amplitude is 20–95 dB.

### 2.12. Network Pharmacology Analysis

Information on FA and AA was retrieved from the PubChem database, and their targets were predicted using the SwissTarget Prediction and Super-PRED databases. The GeneCard human gene database, the DisGenet database, and the OMIM database were then used to obtain target genes for the diseases of interest using “hearing loss” as the search term, with the species set to human. Then, duplicate disease targets were merged and removed. Next, the screened drugs and disease targets were entered into Venny 2.1 software. Then, a protein–protein interaction (PPI) network was constructed using the STRING database, and the biological species was set to “human”. Next, the drug–disease shared targets were imported into DAVID Bioinformatics Resources 6.8 for GO biological process (BP) analysis. Finally, the KEGG pathway enrichment analysis of shared drug targets and disease targets was also performed using DAVID Bioinformatics Resource 6.8.

### 2.13. Docking Analysis

The enrichment of targets in signaling pathways was comprehensively analyzed, and the ability of FA and AA to bind to these targets was analyzed by molecular docking. The SDF structures of FA and AA were downloaded from the PubChem database, and the 3D structures of the core targets were downloaded from the Protein Data Bank (PDB). The Selleck website was used to determine the names of the inhibitors of the core targets, and their SDF structures were downloaded from the PubChem database. Discovery Studio 2016 (DS.2016) software was used to process the obtained 3D structures. The molecular docking of a ligand to a receptor was then performed using a built-in program LibDock. The molecular docking results were visualized as 2D and 3D structure pictures using Discovery Studio Visualization Version 4.5.

### 2.14. Molecular Dynamics Simulation

All-atom molecular dynamics simulations were performed based on the small-molecule and protein complexes that were obtained from the above docking as initial structures, and the simulations were performed using Gromacs 2020.6 software. The small molecules and proteins were described using the CHARMM-36 protein force field. The pdb2gmx subroutine was used to add hydrogen atoms to the system, a truncated cubic TIP3P solvent box was added at a distance of 10 Å from the system, Na^+^/Cl^−^ was added to the system to balance the system charge, and, finally, the topology and parameter files that were used for the simulations were output.

### 2.15. Cellular Thermal Shift Assay (CETSA)

HEI-OC1 cells were treated with FA (200 μM), AA (250 μM), FA+AA (100 μM, 125 μM), or DMSO for 2 h at 37 °C. The cells were harvested in PBS containing 1% protease inhibitor and separated into 10 groups. Individual samples were cooked for 2 min at various temperatures (37–55 °C) using a cycler instrument (Bio-Rad, Hercules, CA, USA). Cell lysates were generated by adding 20 microliters of kinase buffer (CST, Beverly, MA, USA) to the samples and periodically freeze-thawing them in liquid nitrogen. The cell lysates were subjected to immunoblotting with an MMP9 antibody.

### 2.16. Drug Affinity Responsive Target Stability (DARTS)

HEI-OC1 cells were lysed with NP-40 lysis buffer (Shanghai Beyotime Biotechnology Co., Ltd.). The lysates were diluted with 10× TNC buffer (50 mM NaCl, 10 mM CaCl_2_, 50 mM Tris-HCl, pH 8.0) to the same final volume. The proteins were incubated with MMP9 (50, 100, 200, or 400 μM) for 1 h at 4 °C, after which 5 μg/mL streptavidin (Solarbio, Beijing, China) was added to the 1× TNC buffer and incubated for 20 min at room temperature. After 20 min, the reaction was terminated by the addition of Sodium Dodecyl Sulfate loading buffer, and the proteins were detected by immunoblotting with an anti-MMP9 antibody.

### 2.17. Surface Plasmon Resonance (SPR) Analysis

The interaction between MMP9, FA, and AA was detected by SPR using the OpenSPR^TM^ (Nicoya, Costa Rica) at 25 °C. Briefly, recombinant human MMP9 protein (AtaGenix Laboratories, Wuhan, China) was immobilized on the FA and AA Sensor Chip (Nicoya). Gradient concentrations of FA and AA were injected at a flow rate of 20 μL/min in a running buffer [0.05% (*v*/*v*) Tween 20 and 5% (*v*/*v*) DMSO in PBS]. The results were analyzed with the TraceDrawer (Ridgeview Instruments ab, Uppsala, Sweden). Data were fitted to the 1:1 Langmuir binding model, and kinetic parameters were derived.

### 2.18. Infrared Spectroscopy

A Fourier Transform Infrared Spectrometer (FTIR) was used for the detection. The solid sample is finely ground into a powder with a particle size of less than 2.5 microns to prevent infrared light scattering by larger particles. Both FA and AA solids were compressed using the KBr method. The samples were dissolved in DMSO before being analyzed via the thin film method. The infrared spectra were recorded in the range of 4000–400 cm^−1^ with a scanning frequency of 32 times. Data analysis involved identifying functional groups or chemical bonds in the sample by examining the position, intensity, and shape of absorption peaks in the spectrum cross-referenced with the infrared spectral characteristics of known compounds.

### 2.19. Statistical Analysis

The Student’s *t*-test was used to analyze differences between two groups, and one-way ANOVA followed by the Bonferroni post-test was used for comparisons among three or more groups. Two-way ANOVA followed by the Bonferroni post-test was used for comparisons of ABR hearing threshold, threshold shift, and latency of ABR wave I. The rank sum test (Mann–Whitney U test) was used to analyze data that did not conform to a normal distribution. Data are presented as the mean ± SEM. A *p*-value < 0.05 was considered significant (* and # indicate *p* < 0.05, ** and ## indicate *p* < 0.01, *** and ### indicate *p* < 0.001, **** and #### indicate *p* < 0.0001).

## 3. Results

### 3.1. FA Combined with AA Increased the Cell Viability of HEI-OC1 Cells from Cis Damage

**Figure 1:**
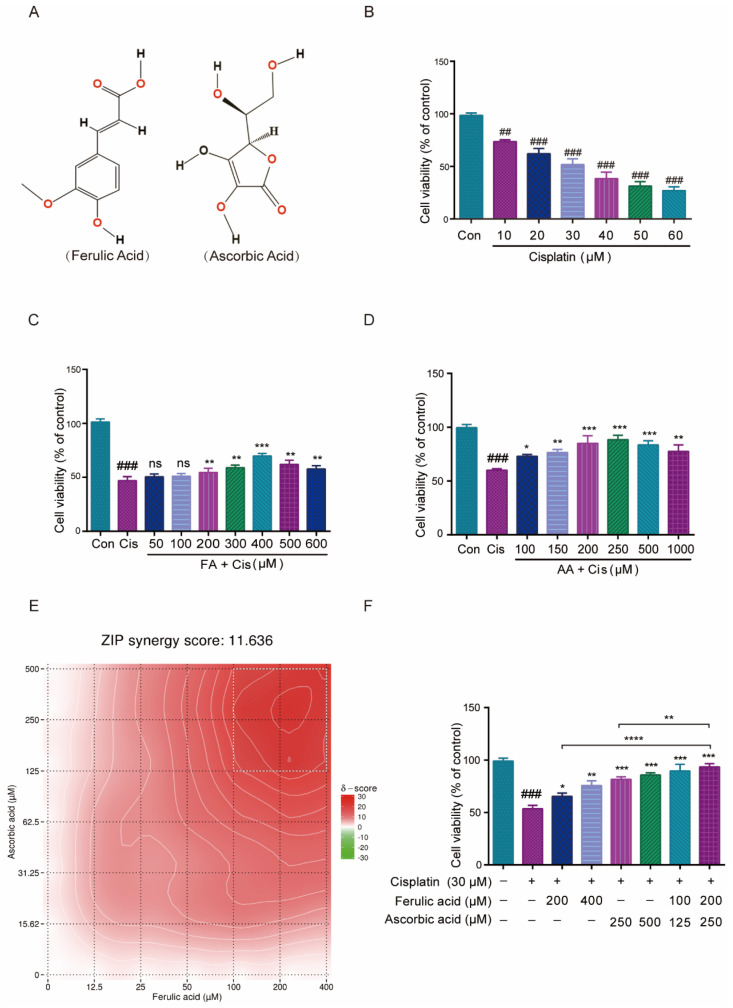
Effect of FA and AA on Cis-induced HEI-OC1 cells’ viability. (**A**) The structure diagram of FA and AA. (**B**) Illustration of changes in cell viability in HEI-OC1 cells post Cis incubation for 24 h; for each group, n = 6. (**C**) Amelioration of the effect of Cis on HEI-OC1 cell viability by FA at different doses; for each group, n = 6. (**D**) HEI-OC1 damage induced by Cis was improved from 100-1000 μm AA; for each group, n = 6. (**E**) Heatmap of drug combination response. Cells were treated with the indicated concentrations of FA, AA, and Cis for 24 h and cell viability was assessed by CCK-8 assay. (**F**) Different doses of FA and AA based on the heat map improved the effect of Cis on HEI-OC1 cell viability; for each group, n = 6. Data are shown as mean ± SEM. Statistical significance is indicated by * *p* < 0.05, ** *p* <0.01, *** *p* < 0.001, **** *p* < 0.0001. ## *p* < 0.01 and ### *p* < 0.001.

The HEI-OC1 cell line serves as a crucial tool in auditory research and is extensively utilized to assess the cytotoxic effects of various drugs on cochlear hair cells [30]. In this work, we first explored how FA and AA would affect HEI-OC1 cell viability. FA and AA are small molecules (Figure 1A) that can dissolve in DMSO. We used infrared spectroscopy to detect the absorption spectra of FA and AA in their solid and liquid phases (in DMSO solution), respectively (Figure S2A,B). We found that FA+AA exhibited properties comparable to those of AA alone, suggesting that the combination of the two entities did not result in any alteration to the intrinsic physical properties of either. In addition, we also examined the effects of FA and AA on HEI-OC1 cell survival at different concentrations, and there was no statistically significant difference between FA at concentrations lower than 600 μM, and AA did not show any effect on cell viability in the range of concentrations we chose (Figure S2C,D). To examine the effect of Cis on cell viability, HEI-OC1 cells were treated with Cis at varying concentrations (0, 10, 20, 30, 40, 50, or 60 μM) for 24 h, and the cells were collected and analyzed using the CCK8 technique. As shown in Figure 1B, Cis-stimulated HEI-OC1 cells had considerably decreased viability compared to normal cells in a Cis-dose-dependent manner. Similarly, a single intraperitoneal injection of Cis (30 mg/kg) was administered. After 7 days, the mice exhibited varying but significant loss of cochlear hair cells, as illustrated in Figure S1. To assess the potential protection effect of FA, HEI-OC1 cells were pretreated with FA doses ranging from 50 to 600 µM for 2 h before being stimulated with 30 µM Cis for 24 h. FA pretreatment significantly improved HEI-OC1 viability in a dose-dependent manner (Figure 1C).

Similarly, the pretreatment of HEI-OC1 cells with different concentrations of AA (100–1000 µM) for 2 h dramatically enhanced HEI-OC1 cell viability in a dose-dependent manner (Figure 1D). The online SynergyFinder software (https://synergyfinder.fimm.fi, accessed on 20 January 2024) was used to calculate drug synergy scoring with the “viability index” by the response surface model and zero interaction potency (ZIP) calculation method and found that two concentration combinations (FA200 μM + AA250 μM) achieved the highest score (Figure 1E, with white rectangles representing areas of highest synergy). The ZIP Synergy Score is a metric used to evaluate the interaction between two drugs or compounds in a combination treatment. A score of 11.636 is a positive value, indicating that the combination of the two drugs has a synergistic effect. This result suggests that the combined effect of the drugs is significantly greater than what would be expected if they acted independently. We then chose these two combinations (FA100 μM + AA125 μM and FA200 μM + AA250 μM) to pretreat HEI-OC1 cells for 2 h, and the results showed considerable increases in cell viability (Figure 1F). The cell viability of the FA200 μM + AA250 μM group was higher than that of FA200 μM and AA250 μM alone. At the same drug concentration 6 h earlier, neither FA, AA, nor the combination of FA + AA demonstrated a significant protective effect (Figure S2G). The combined application of FA and AA ameliorates Cis-induced HEI-OC1 cytotoxicity to a greater extent than either agent alone.

### 3.2. FA, AA, and FA+AA Treatments Protected HEI-OC1 Cells from Cis-Related Damage

**Figure 2:**
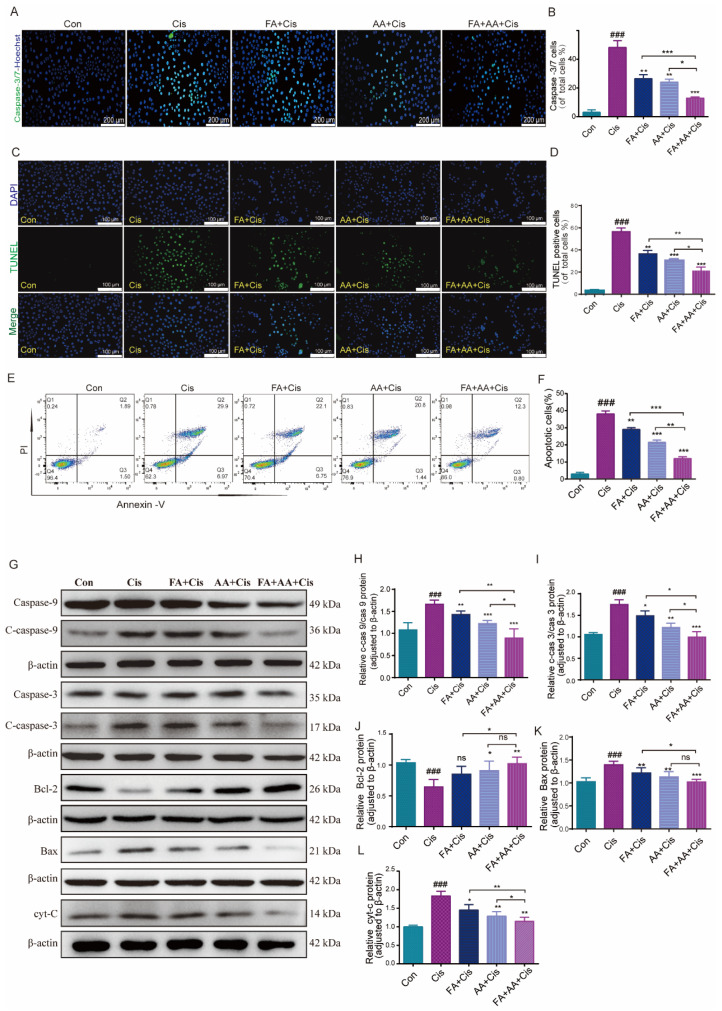
FA combined with AA protected against Cis-induced damage. (**A**,**B**) Representative images of caspase-3/7 staining in HEI-OC1 cells; for each group, n = 3. Scale bars = 200 μm. (**C**) Representative images of TUNEL staining in HEI-OC1 cells. Scale bars = 100 μm. (**D**) Quantification of TUNEL-positive cells of total cells; n = 3. (**E**,**F**) Flow cytometry analysis of FA and AA on cell apoptosis induced by Cis. Apoptotic cells (early apoptosis and late apoptosis) were detected by Annexin V-FITC/PI kit; for each group, n = 3. (**G**–**L**) Western blot analysis of apoptosis-related proteins; for each group, n = 6. Data are shown as mean ± SEM. * *p* < 0.05, ** *p* < 0.01, *** *p* < 0.001; ### *p* < 0.001.

To test whether FA, AA, and FA+AA affected cell proliferation, we performed the Hoechst fluorescence assay. As illustrated in Figure S2E,F, we assessed the number of cells in each group from 0 to 24 h. It is evident that, after 24 h, there is no significant difference in cell counts among the Con, FA, AA, and FA+AA groups. However, the number of cells in the Cis group is notably lower than in other groups. Next, we evaluated the effects of FA, AA, and FA+AA on the apoptosis of HEI-OC1 cells using caspase-3/7 and TUNEL staining. Using Hoechst as an internal reference, the caspase-3/7 fluorescence signal was significantly enhanced in the Cis group and differentially attenuated in the FA+Cis, AA+Cis, and FA+AA+Cis groups (Figure 2A,B). Similarly, in TUNEL staining with DAPI as an internal reference, similar to the caspase-3/7 fluorescence assay, TUNEL staining attenuation was most pronounced in the FA+AA+Cis group (Figure 2C,D). Subsequently, these drugs’ effects on apoptosis in HEI-OC1 cells were further confirmed using flow cytometry with Annexin V-FITC/PI labeling. As shown in Figure 2E,F, the proportion of apoptotic cells (early apoptosis and late apoptosis) was significantly greater in the Cis group compared to the control group (36.87% vs. 3.39%). However, after Cis was added to the treatment with FA, AA, and FA+AA, the percentage of apoptotic cells decreased to 28.85%, 22.24%, and 13.1%, respectively. We also assessed the levels of apoptosis-related proteins, including Bax, Bcl-2, and cyt-C and caspase-3, caspase-9, and their cleaved forms, in the HEI-OC1 cells using Western blot analysis. We found that FA, AA, and FA+AA significantly regulated the levels of these proteins. More importantly, proteins closely associated with apoptosis such as Bax and cyt-C expression were down-regulated, and Bcl-2 expression was up-regulated after FA, AA, and FA+AA treatment (Figure 2G–L). In conclusion, these results indicated that FA, AA, and FA+AA reduced Cis-induced apoptosis in HEI-OC1 cells.

### 3.3. FA, AA, and FA+AA Treatments Reduced Cis-Induced Apoptosis by Lowering ROS Levels

**Figure 3:**
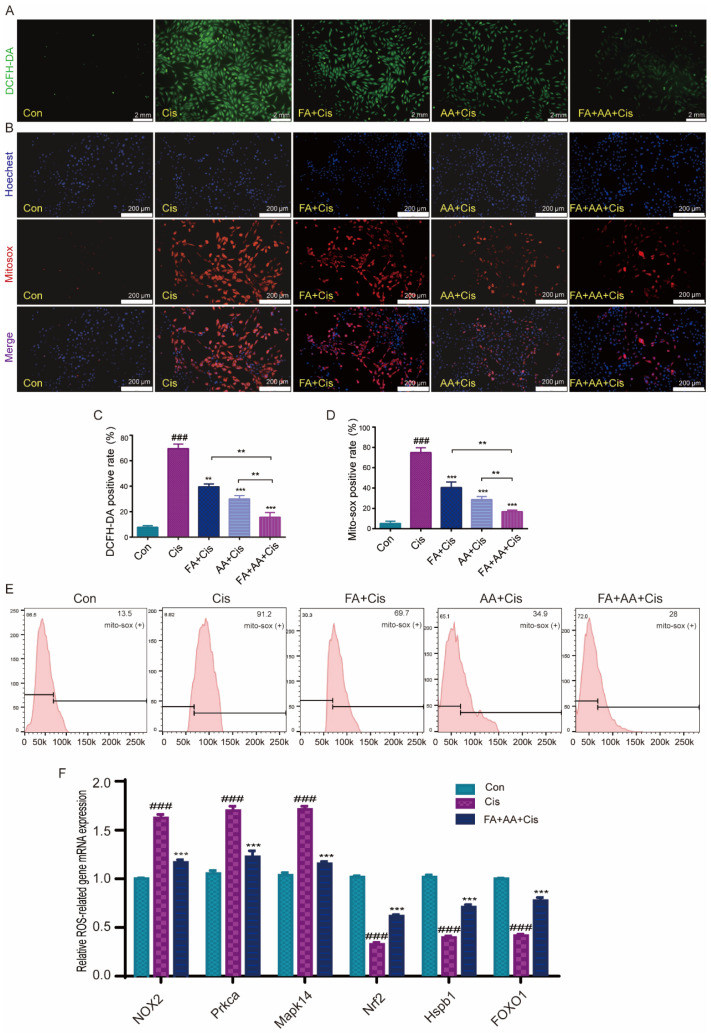
Effects of FA, AA, and FA+AA on ROS production in HEI-OC1 cells after Cis injury. (**A**) The intracellular ROS level was measured using a peroxide-sensitive fluorescent probe, DCFH-DA. Scale bars = 2 mm. (**C**) Representative quantitative changes in DCFH-DA in HEI-OC1 cells from each group; for each group, n = 3. (**B**,**E**) Representative images and quantitative changes in Mitosox-red in HEI-OC1 cells from each group; for each group, n = 3. Scale bars = 200 μm. (**D**) Representative quantitative changes in Mitosox-red in HEI-OC1 cells from each group (flow cytometry detection); for each group, n = 3. (**F**) Using qPCR analysis, we examined six ROS-related gene expression levels (NOX2, Prkca, Mapk14, Nrf2, Hspb1, and FOXO1) to validate the increased generation of ROS. In our analysis, GAPDH is a housekeeping gene for normalization; for each group, n = 3. Results represent the mean of three independent experiments ± the mean of SEM experiments. ** *p* < 0.01, *** *p* < 0.001. ### *p* < 0.001.

ROS levels are an important sign of oxidative stress in cochlear hair cells, and they play an important role in Cis-induced damage [31]. To investigate whether FA, AA, and FA+AA ameliorate Cis-induced cell apoptosis by regulating ROS levels, we examined the intracellular ROS levels in HEI-OC1 cells using the fluorescent probe DCFH-DA, a cell-permeable probe specific for the detection of intracellular ROS. As expected, Cis-treated HEI-OC1 cells had higher levels of intracellular ROS than the control group. Interestingly, pretreatment with FA, AA, and FA+AA resulted in a significant reduction in ROS (Figure 3A,C). We then used MitoSOX fluorescence and flow cytometry to further detect the mitochondrial superoxide. The results confirmed that FA, AA, and FA+AA all reduced mitochondrial ROS (Figure 3B,D,E). Next, we validate the enhanced production of ROS by examining six ROS-related gene expression levels using qPCR analyses, including *NOX2*, *Prkca*, *Mapk14*, *Nrf2*, *Hspb1*, and *FOXO* (Figure 3F). The upregulation of *NOX2*, *Prkca*, and *Mapk14* genes generally indicates an enhanced production of ROS and activation of signaling pathways related to stress response, inflammation, and immune activation. The downregulation of *Nrf2*, *Hspb1*, and *FOXO* genes collectively suggests a weakened cellular defense against oxidative stress and other stressors. In the FA+AA+Cis group, we found that the expression of these six genes showed varying degrees of change. In conclusion, the findings indicate that the reduction in ROS in HEI-OC1 cells is a key factor underlying the protective effect of FA+AA on cells injured by Cis.

### 3.4. FA+AA Enhanced the Outer Hair Cell Survival in the Cultured Cochlear Explants and In Vivo

**Figure 4:**
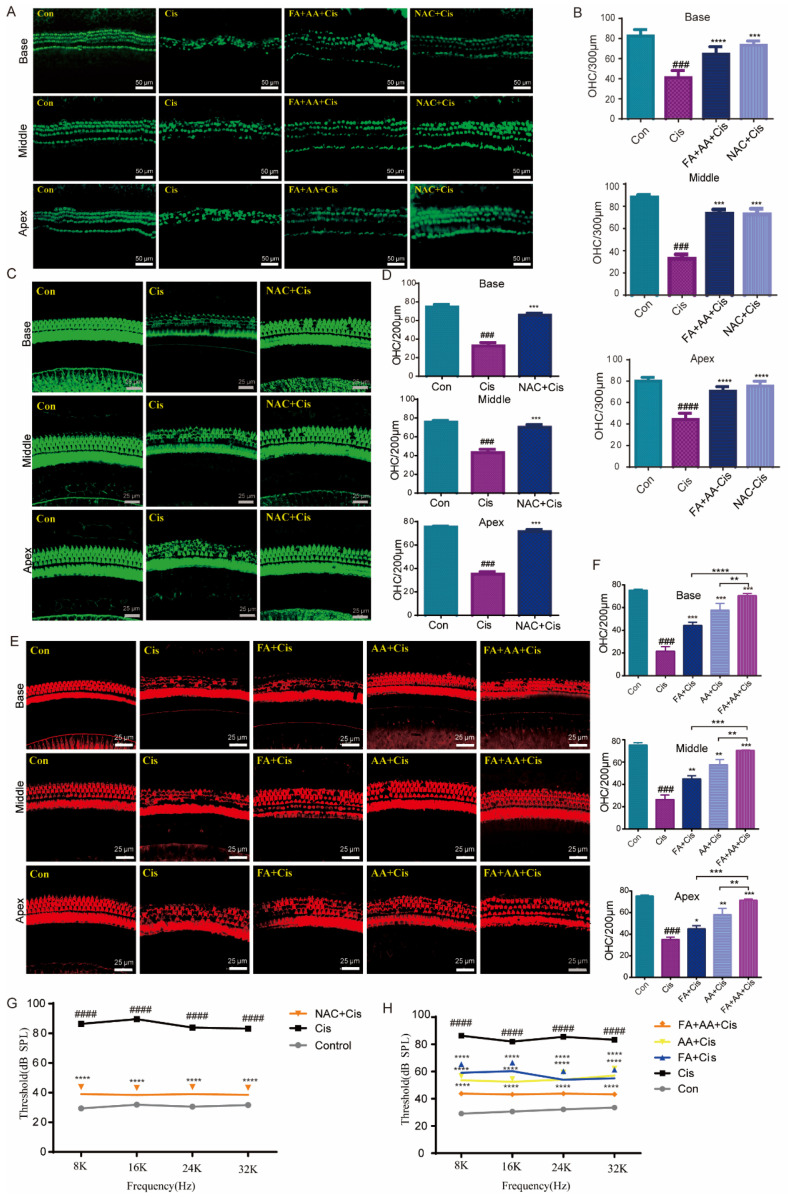
FA and AA are protective in explants and in vivo. (**A**) Representative immunofluorescence photograph showing the apex, middle, and base regions of the cochlear explants treated with medium-alone Con, Cis, and FA+AA+Cis for 24 h, and immunolabeled for myosin7a (green). Scale bars = 50 μm. (**B**) The illustration of OHCs counting (myosin7a, green) (n = 3). (**C**) Immunostaining with Alexa Fluor 594 phalloidin in the apex, middle, and base turns of cochleae from different groups in vivo. Scale bars = 25 μm. (**D**) The illustration of OHCs counting; for each group, n = 6. (**E**) Immunostaining with Alexa Fluor 488 phalloidin in the apex, middle, and base turns of cochleae from different groups in vivo. Scale bars (25 μm). (**F**) The illustration of OHCs counting; for each group, n = 6. (**G**) ABR thresholds at 8, 16, 24, and 32 kHz were measured 14 days after the injection of saline (Con), cisplatin 30 mg/kg (Cis), or cisplatin plus 150 mg/kg NAC (NAC+Cis) (each group, n = 6 mice). (**H**) ABR thresholds at 8, 16, 24, and 32 kHz were measured 14 days after the injection of saline (Con), cisplatin 30 mg/kg (Cis), or cisplatin plus 50 mg/kg FA + 75 mg/kg AA (FA+AA+Cis) (each group, n = 6 mice). The data represent mean ± SEM; * *p* < 0.05, ** *p* < 0.01, *** *p* < 0.001, **** *p* < 0.0001; ### *p* < 0.001, #### *p* < 0.0001.

Next, we used cultivated cochlear explants as a model to assess the protective effects of FA, AA, and FA+AA. Rescue experiments were performed using the antioxidant NAC [32]. Mice cochlear explants were collected from newborn pups (3–4 days old) and cultured in vitro with chemical treatments (Cis 30 μM, FA200 μM, AA 250 μM, NAC 2 mM). NAC is a scavenger of ROS. As shown in Figure 4A, the in vitro culture had a characteristic three row structure of outer hair cells along the cochlear explants. Cis treatment caused significant shedding of outer hair cells. In contrast, NAC and FA+AA dramatically boosted the amount of hair cells that survived in the explants (Figure 4B). For in vivo analysis, C57BL/6 mice (both sexes) were injected with 100 mg/kg (FA), 150 mg/kg (AA), or 50 mg + 75 mg/kg (FA+AA) 2 h before Cis injection (i.p., 30 mg/kg). The control group comprised eight animals that did not receive any injection. The ABR was measured after 14 days. In comparison to the control group, animals treated with Cis lost a large number of cochlear outer hair cells at the base, middle, and apex. In vivo, the application of NAC was found to exhibit antioxidant action and boost cochlear hair cell survival (Figure 4C,D). When FA, AA, and FA+AA were administered before Cis injection, the quantity of cochlear outer hair cell (OHCs) stores increased in all three groups revealed by immunostaining with Alexa Fluor 594 phalloidin (Figure 4E,F). The ABR test demonstrated that the FA, AA, and FA+AA groups all had varied degrees of considerably reduced threshold elevation, with the FA+AA group having comparable hearing thresholds with the NAC group (Figure 4G,H). All mice were confirmed to have normal hearing by ABR testing before treatment (baseline threshold: 20–30 dB SPL, test frequency 8–32 kHz). Furthermore, applying FA+AA to the middle ear cavity 2–4 h in advance, as demonstrated in Figure S4A,B, improved the number of hair cell survivors. In conclusion, FA+AA can prevent Cis-induced damage to cochlear OHCs in vivo.

### 3.5. Network Pharmacology and Transcriptomics Analysis Identified the MAPK Pathway as a Potential Mechanism for FA+AA Protective Activity

**Figure 5:**
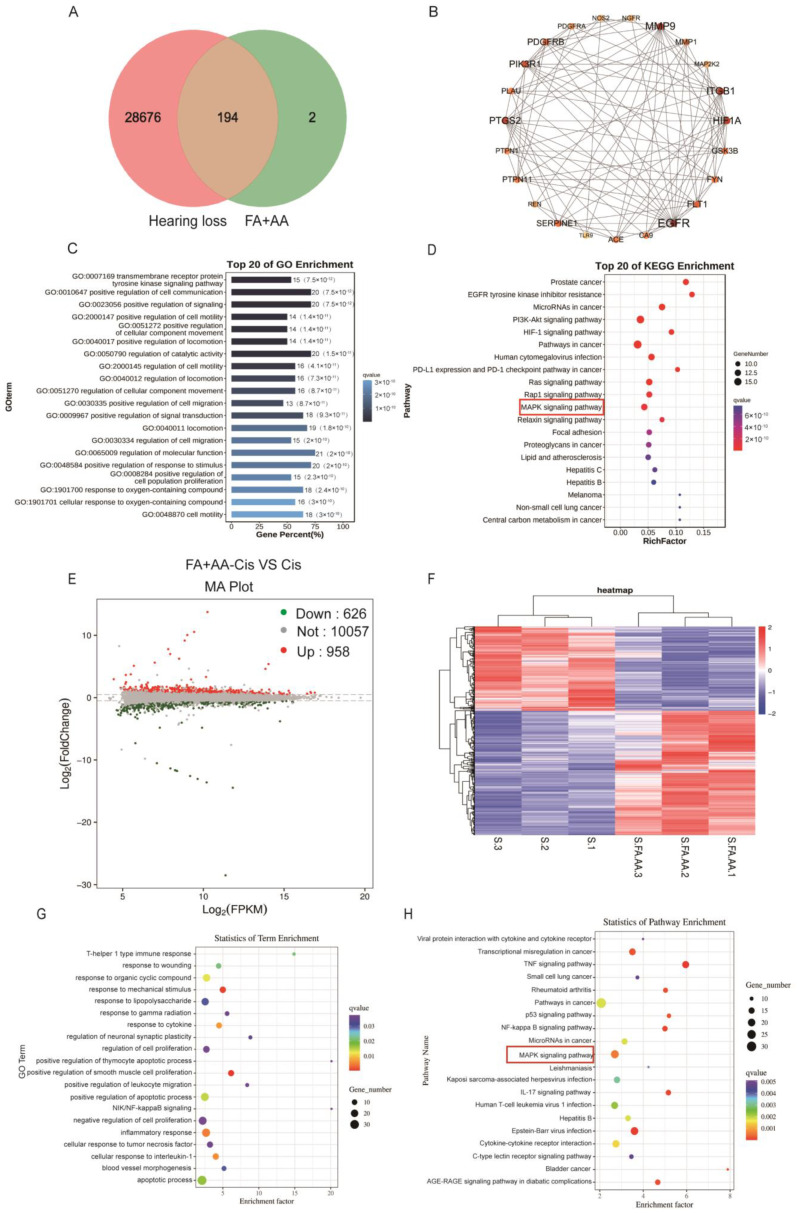
Results of network pharmacological and transcriptomic analyses. (**A**) Venn diagram showing the number of common targets of FA and AA associated with hearing loss. (**B**) Protein–protein interaction (PPI) network of common targets analyzed by Cytoscape software based on degree values. (**C**,**D**) GO and KEGG analyses of related signaling pathways between shared targets. (**E**) Volcano plot showing dysregulated genes between the Cis and FA+AA+Cis groups. (**F**) Heatmap depicting differential gene expression in FA+AA+Cis-treated HEI-OC1 cells versus Cis-treated HEI-OC1 cells. The three Cis-treated groups are represented by S1, S2, and S3, and three FA+AA+Cis-treated groups are represented by S. FA.AA1, S.FA.AA2, and S.FA.AA3. (**G**,**H**) GO- and KEGG-based gene function analysis showing the top 20 pathways most affected by FA+AA treatment in HEI-OC1 cells.

We aim to explore the potential mechanism for FA and AA by using network pharmacology. To this end, FA and AA were separately searched using the PubChem database (https://pubchem.ncbi.nlm.nih.gov/, accessed on 21 October 2023); the SMILE numbers were entered into the SwissTarget prediction database (http://www.swisstargetprediction.ch/, accessed on 25 October 2023), and the Super-PRED (https://prediction.charite.de/index.php, accessed on 1 November 2023) database predicted a total of 196 targets. The GeneCard human gene database, DisGenet database (https://www.disgenet.org/), and OMIM database (https://omim.org/) were used to conduct target searches using “hearing loss” as the search term. The species was set to “human” to obtain the disease target genes. Disease targets that were retrieved from the databases were merged and duplicates removed, and a final list of 28,870 disease targets was obtained. The screened drug targets and disease targets were entered into the Venn diagram creation software Venny 2.1, and 194 shared targets were obtained (Supplementary Materials); these targets were used as predicted targets for the effects of drugs on diseases in subsequent analyses, as shown in Figure 5A. A PPI network was constructed with these 194 potential targets using the String database. The first 22 of these targets were visualized with Cytoscape 3.7.2 according to the degree, as shown in Figure 5B. To determine the possible mechanisms by which FA+AA ameliorates Cis ototoxicity, 194 predicted targets were subjected to GO and KEGG enrichment analyses. The top 20 major KEGG pathways that were involved in the FA+AA-mediated amelioration of Cis ototoxicity were identified based on *p* values (*p* < 0.05) and count values (Figure 5C,D). These included MAPK signaling pathways that are involved in important biological responses, such as cell proliferation, differentiation, and apoptosis. Next, we performed the transcriptome sequencing of the FA+AA+Cis and Cis groups to further identify potential target genes regulated by FA+AA treatment in HEI-OC1 cells, and the results are shown in Figure 5E,F; RNA-seq analyses identified 1584 differentially expressed genes (958 upregulated and 626 downregulated). GO and KEGG analyses were performed to elucidate the most possible signaling pathway. As shown in Figure 5G,H, the MAPK signaling pathway interested us most in the top 20 altered signaling pathways, as this pathway was also enriched in our network pharmacology analysis (Figure 5D).

### 3.6. FA and AA Could Interact Directly with MMP9

**Figure 6:**
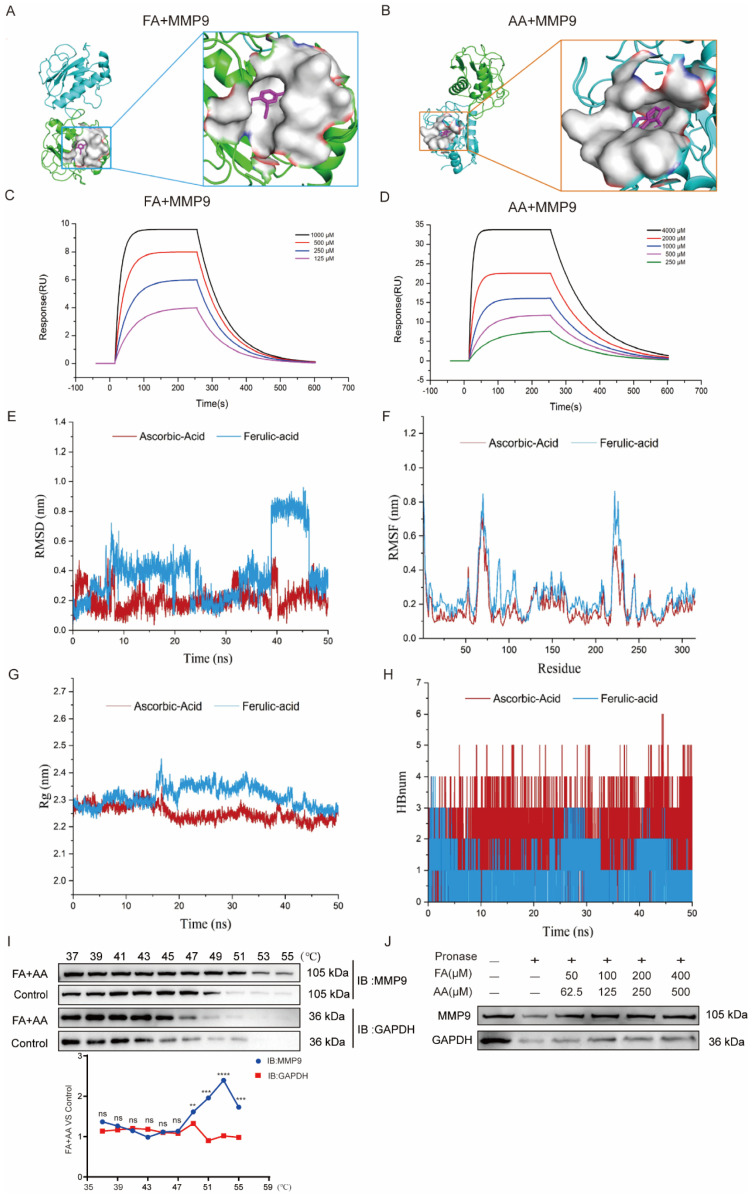
FA and AA are two small-molecule substances with which MMP9 interacts directly. (**A**) Molecular docking pattern of FA and MMP9. (**B**) Molecular docking pattern of AA and MMP9. (**C**) SPR analysis of FA binding to MMP9. (**D**) SPR analysis of AA binding to MMP9. (**E**–**H**) All-atom molecular dynamics simulations were performed based on the small molecules and protein complexes obtained by the above docking as initial structures, and the simulations were carried out using Gromacs 2020.6 software for subsequent analyses of RMSD, RMSF, Rg, and hydrogen bonding. (**I**) FA+AA promoted MMP9 resistance to different temperature gradients (CETSA); for each group, n = 6. (**J**) FA+AA promotes MMP9 resistance to proteases (DARTS); for each group, n = 6. ** *p* < 0.01, *** *p* < 0.001 and **** *p* < 0.001.

As we enriched MMP9 by network pharmacology, we further performed molecular docking for interaction between FA and AA with MMP9. As shown in Figure 6A,B, FA could interact with MMP9 via hydrophobic contacts with residues Leu 418, Leu 397, and Arg 424 as well as hydrogen bonds with Glu 402, and carbon–hydrogen bonds with Pro 415 and Met 422. AA established hydrogen connections with the MMP9 residues Arg 424, Met 422, Tyr 420, and Ala 417 (Figure S3). FA and AA displayed binding affinities of −7.0 kcal/mol and −6.6 kcal/mol for MMP9. Furthermore, surface plasmon resonance (SPR) investigations revealed that FA and AA could bind directly to MMP9 (Figure 6C,D). All-atom molecular dynamics simulations were performed using the beginning structures of the small molecules and protein complexes generated by the aforementioned docking methods (see Section 2). The simulations were run with Gromacs 2020.6 software, and further analyses such as Root Mean Square Deviation (RMSD), Root Mean Square Fluctuation (RMSF), radius of gyration (Rg), and hydrogen bonding were performed (Figure 6E–H). The RMSD value of AA remained between 0.1 nm and 0.4 nm throughout the simulation process. For FA, the small molecules in the first 38 ns of the simulation are similar to AA, always remaining bound to the protein-binding cavity and shaking. After maintaining stable binding in the new binding cavity (RMSD = 0.8 nm) for a period of time (38 ns–46 ns), small molecules spontaneously migrate to the original protein-binding cavity again under the influence of the surrounding environment and thermal motion and maintain stable binding until the end of the simulation, indicating that the previous binding cavity is still the dominant binding conformation of FA (Figure 6E). RMSF analysis demonstrated structural adaptability. As shown in Figure 6F, there was a certain peak in the protein RMSF curve during the entire simulation process. Overall, the RMSF of the FA complex is higher than that of AA. Combined with the RMSD analysis, this result may reflect the detachment of FA from the original protein-binding cavity during the simulation process, which has a certain impact on the flexible residues of the protein. Next, we conducted an Rg analysis to characterize the compactness of protein structures (Figure 6G). The results were consistent with the RMSD and RMSF analyses results, with the average Rg value of FA higher than that of AA. Overall, the Rg curves of the two complex proteins tended to stabilize with the simulation, indicating that the complex gradually stabilized. Based on the HBond plot and the previous analysis, it can be concluded that AA and FA have always formed a certain number of hydrogen bonds with proteins during the simulation process (Figure 6H). Among them, AA maintains a relatively stable number of 3–5 hydrogen bonds in the later stage of the simulation. Although FA forms fewer hydrogen bonds than AA, it still maintains at least one hydrogen bond with the protein during the simulation process, and its binding is relatively stable. The overall stability of the AA complex was superior to that of the FA complex, with substantially stronger binding. Furthermore, a CETSA [33,34] demonstrated that FA+AA improved MMP9 thermal stability (Figure 6I). We evaluated the stability of MMP9 and GAPDH. FA and AA can enhance thermal stability, even at temperatures exceeding 50 °C, a property that is diminished in control samples lacking FA and AA. We discovered that there was little variation in the degradation temperature between MMP9 and GAPDH in the control group. A DARTS [34,35] experiment revealed that, even at lower concentrations of FA+AA, MMP9 is well protected from protease degradation, suggesting that FA+AA could stabilize MMP9 and enhance its resistance to protein hydrolysis in the assay (Figure 6J). These results suggest that FA and AA could bind to MMP9 directly and, thus, regulate its protein level.

### 3.7. FA+AA Reduces Cis-Induced Damage Through the Inhibition of MMP9 and p38MAPK Signaling Pathways

**Figure 7:**
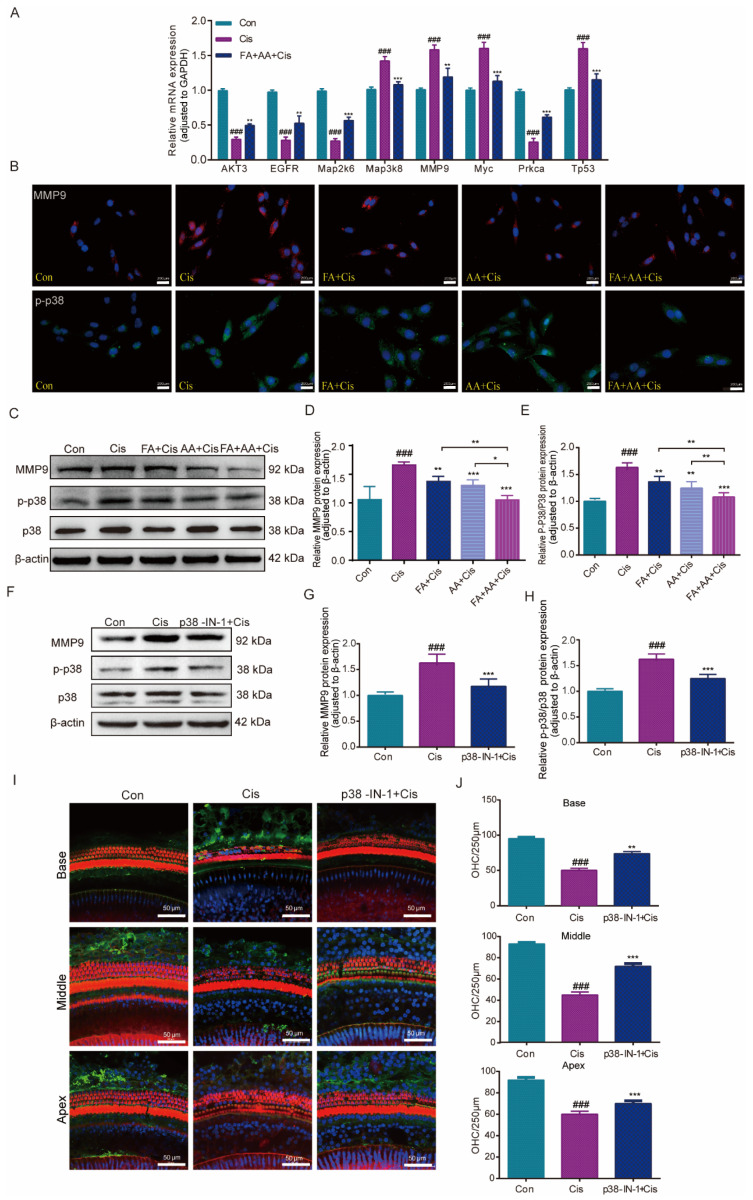
Effects of FA+AA on the MMP9 and MAPK pathway during Cis-induced damage. (**A**) Relative mRNA levels of these genes were normalized to GAPDH levels in HEI-OC1 cells treated with Cis or FA+AA+Cis, as determined by qRT-PCR; for each group, n = 3. (**B**) Immunofluorescence analysis demonstrated the impact of FA and AA on the expression of MMP9 and p-P38 in HEI-OC1 cells. Scale bars = 200 μm. (**C**–**E**) Effect on MMP9, P38, and p-P38 protein expression after the administration of FA+AA to HEI-OC1 cells, as determined by Western blotting analysis; for each group, n = 6. (**F**–**H**) Relative expression levels of MMP9, P38, and p-p38 were determined by Western blotting analysis; for each group, n = 5. (**I**) Immunofluorescence showed that p38-MAPK-in-1 ameliorated Cis-induced hair cell damage in vivo. (**J**) The illustration of cochlear OHCs counting (red) (each group, n = 3). The data represent the mean ± SEM; * *p* < 0.05, ** *p* < 0.01, *** *p* < 0.001. ### *p* < 0.001.

To corroborate the RNA-seq data, eight MAPK-signaling-pathway-related genes, including MMP9, were chosen to be measured by qPCR for their expression. The results confirmed that the mRNA expressions of *Map3k8*, *MMP9*, *Myc*, and *Tp53* were elevated by Cis administration, whereas they decreased in the FA+AA+Cis group. *Akt3*, *Egfr*, *Map2k6*, and *Prkca* were downregulated in the Cis group and only slightly increased in the FA+AA+Cis group (Figure 7A). *Akt3*, *Egfr*, *Map2k6*, and *Prkca* regulate biological functions such as cell survival and metabolism, cell proliferation, stress response, and cell invasion. P38 is an important member of the MAPK family. Furthermore, we investigated the effects of FA, AA, and FA+AA on the expression of MMP9 and p-p38 in HEI-OC1 cells. As illustrated in Figure 7B–E, FA, AA, and FA+AA reduced the levels of these two proteins to varying degrees. In particular, both MMP9 and p-p38 protein expression were lowest in the FA+AA group. We wanted to know if inhibiting the p38 signaling pathway can have a protective effect. Next, we evaluated the levels of p38 protein in the Cis, P38 MAPK-IN-1+Cis, and Con groups to determine the impact of these treatments on the MAPK signaling pathway (Figure 7F–H). In the Cis group, the expression of MMP9 and p-p38 proteins was increased. Our qPCR data showing the downregulation of *MMP9* mRNA aligns with observed reductions in MMP9 protein and preserved hair cell counts, supporting a potential transcriptional repression mechanism for FA+AA-mediated protection. These data suggest that the protective effects of FA+AA against Cis-associated ototoxicity may involve, at least in part, the inhibition of the p38 MAPK signaling and MMP9. Pretreatment with the MAPK inhibitor P38 MAPK-IN-1 (1 mg/kg, i.v.) two hours before Cis treatment enhanced the number of cochlear outer hair cell survivors in both apex, middle, and base structures (Figure 7I,J). In the FA+AA treatment group, we performed immunofluorescence staining on mouse cochlear hair cells and observed the expression of MMP9 protein in the outer hair cells (Figure S4C). MMP9 was predominantly localized to the cytoplasm of OHCs. Co-treatment with FA+AA reduced the overall expression of MMP9 and decreased cytoplasmic accumulation. These data imply that FA+AA’s protective impact against Cis-associated ototoxicity is at least largely through the inhibition of p38MAPK signaling and MMP9.

**Figure 8:**
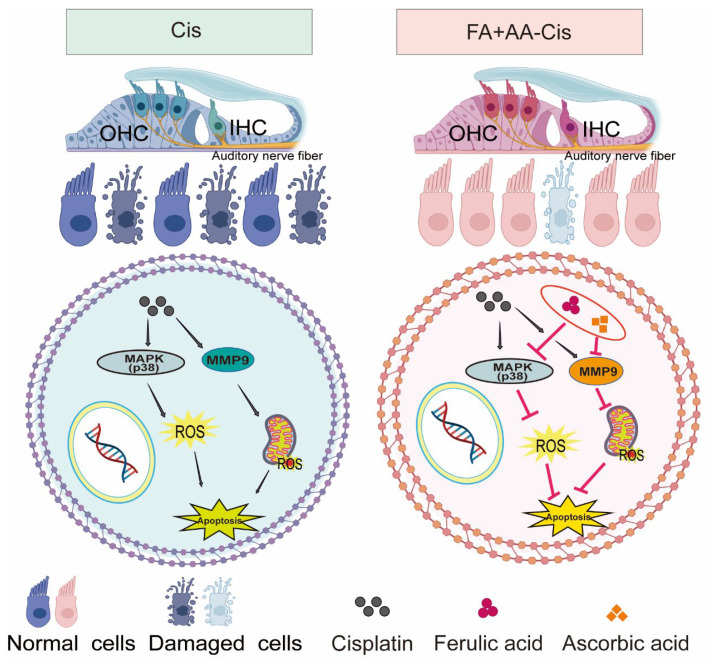
The mechanism overview of FA + AA.

As illustrated in Figure 8, FA + AA may exert its protective effects through two potential mechanisms: (1) the inhibition of p38 MAPK signaling, leading to reduced MMP9 expression, and (2) the direct or indirect regulation of MMP9 activity. These mechanisms are not mutually exclusive and may work in concert to mitigate Cis-induced ototoxicity.

## 4. Discussion

Platinum-based chemotherapeutic agents Cis and carboplatin are widely used in cancer treatment worldwide and may result in ototoxic hearing loss [36]. Cis has significant ototoxic effects, constituting a global public health risk. Numerous tactics for reducing or reversing the ototoxic effects of Cis have been tested, but none have exceeded the effectiveness standards required for regulatory approval. The FDA approved sodium thiosulfate for use in pediatric children with localized nonmetastatic solid tumors at a lower dose [37]. The systemic use of sodium thiosulfate may be an interesting area of study, particularly in the context of “combination antioxidant therapy” (multi-targeted medication combinations), which has been found to activate several biochemical pathways [38,39].

The “combination antioxidant therapy” strategy inspired the exploration of the combinatorial application of Cis with antioxidants, which could inhibit ROS; the latter is known as an important mediator of ototoxicity, and the reduction in ROS has a causal relationship with the improvement of survival ability. However, these techniques are still in preclinical investigations, and additional research is required to evaluate their efficacy [37]. One of the promising antioxidants is FA, which has been demonstrated to possess a range of beneficial physiological functions, including antioxidant, anti-inflammatory, antimicrobial, and anticancer properties with relatively low toxicity [40]. FA protects against Cis-induced cytotoxicity by preventing Cis-induced ROS production and inducing endogenous antioxidant production, suggesting that FA may protect against Cis-induced ototoxicity [24]. Another candidate worthy of examination is AA. AA protects against ototoxicity brought on by Cis. In addition, the intracameral application of AA in the tympanic cavity does not exert toxic effects on the cochlea [41]. AA increased the survival number of HEI-OC1 cells by inhibiting apoptosis activation due to neomycin-induced ROS production and attenuating cellular damage. AA inhibited apoptosis by suppressing JNK activation and p38 phosphorylation [42]. Previous research has shown that the combination of FA and AA has a synergistic protective effect against alleviating isoproterenol (ISO)-induced cardiac necrosis in rats [43]. In this study, we confirmed the low toxicities of FA, AA, and the combination of FA and AA. Specifically, Bax is a pro-apoptotic protein that promotes mitochondrial outer membrane permeabilization, leading to the release of cyt-C and the activation of the caspase cascade. In contrast, Bcl-2 is an anti-apoptotic protein that inhibits Bax activity, thereby preventing apoptosis. Caspases, particularly caspase-3, are executioner proteases that mediate the cleavage of cellular substrates, resulting in the morphological and biochemical changes characteristic of apoptosis. In our study, we examined the expression levels of these proteins to assess their involvement in the apoptotic response induced by Cis. We showed that the administration of FA, AA, or FA+AA significantly reduced ROS levels in Cis-treated HEI-OC1 cells, influenced the expression of apoptosis-related proteins, and decreased the apoptosis rate. Increases in the number of cochlear hair cell survival by these treatments were also observed in in vivo experiments in mice. More importantly, the FA+AA combination always showed a superior protective effect than AA or FA alone on Cis-induced cell death. The ineffectiveness of 6 h FA+AA pretreatment highlights a critical time window for protection. This situation may reflect the rapid oxidization of FA and AA and the loss of their antioxidant capacity after exposure. Future research should determine the minimum effective pretreatment time and explore adjuvant therapies that immediately inhibit ROS.

Apoptosis is the primary form of cell death triggered by Cis in the cochlea [44]. Cis-induced apoptosis is principally attributed to oxidative stress and the breakdown of oxidized metabolites in mitochondria [45], leading to free radical buildup. Excessive free radical generation can disrupt cellular redox equilibrium and cause apoptosis [46]. Furthermore, it promotes lipid peroxidation and suppresses the production of endogenous antioxidants. Maintaining mitochondrial activity and inhibiting free radical buildup by the mitochondrial apoptotic pathway is critical for minimizing Cis-induced damage. In this study, we utilized a HEI-OC1 cell model to examine the anti-apoptosis effects of these antioxidants. Our findings demonstrated a large increase in TUNEL-positive cells in the Cis group, implying that Cis causes hair cell loss primarily via triggering apoptosis, which supports prior findings. Interestingly, FA+AA treatment reduced the number of lost cells, and TUNEL-positive cells decreased significantly. In vivo, tests revealed that FA+AA enhanced mice’s hearing and increased OHCs survival by 60% relative to Cis-only treatment. In vivo and in vitro studies revealed that FA and AA might combinedly inhibit Cis-induced cochlear hair cell loss in mice. The concentration of Cis used in this study was chosen to induce significant ototoxicity within a short timeframe, enabling a clear evaluation of the protective effects of FA and AA. While this model does not fully replicate the progressive hearing loss observed in patients, it provides a robust platform for assessing the efficacy of therapeutic interventions. The observed hearing loss may not solely be attributed to OHCs loss, as Cis is known to cause damage to other cochlear structures, including the stria vascularis, spiral ganglion neurons, and synaptic connections. These additional mechanisms may explain the severity of hearing loss despite the limited OHCs loss observed in some regions.

Our findings indicate that FA+AA co-treatment attenuates Cis-induced hearing loss observed at 7 days post administration. However, the reliance on single-timepoint ABR assessment at this early stage represents a significant methodological constraint. Notably, cisplatin exhibits prolonged retention in cochlear tissues (stria vascularis and organ of Corti) for weeks to months, where it perpetuates oxidative stress and inflammatory cascades even after systemic clearance. This persistent ototoxic activity raises critical questions about whether the observed protective effects of FA+AA reflect genuine long-term preservation or merely the transient mitigation of hearing threshold shifts. To address these limitations, longitudinal ABR evaluations and histological analysis at 7, 14, and 28 days post Cis exposure are required to resolve (1) whether FA+AA’s protective effects endure beyond acute injury (7 days) or wane as Cis retention drives secondary damage, and (2) whether delayed treatment with FA+AA could rescue hearing function, which would be clinically relevant for patients receiving multi-cycle chemotherapy.

To investigate the mechanism behind FA+AA’s protective effect against Cis-associated cell injury, we used network pharmacology and transcriptomics to explore the potential pathway. In total, 1584 differentially expressed genes between the Cis and FA+AA+Cis groups were identified by RNA-seq. Of these, 958 genes were elevated and 626 genes were downregulated. KEGG analysis revealed that the identified significantly differentially expressed genes were primarily involved in apoptosis-related pathways, including the MAPK signaling pathway, the TNF signaling pathway, the p53 signaling pathway, and the NF-kappa B pathway, implying that FA+AA may protect HEI-OC1 cells from Cis-induced injury via a complex mechanism.

Cyberpharmacology is a bioinformatics strategy emerging as a novel research methodology for finding potential targets and pharmacological mechanisms [47]. We investigated the MAPK signaling pathway and focused on MMP9. MMP9 is a modulator of caspase-3 activation and is necessary for apoptosis. The inhibition of MMP9 improved cell survival and decreased apoptosis induced by hyperglycemia [48]. Our molecular docking analysis demonstrated that FA and AA bound to MMP9 in different structural domains, while AA may have a higher affinity than FA, which was confirmed in late molecular dynamics simulations. CETSA and DARTS experiments revealed that these two compounds enhance the stability of the MMP9 protein. These findings strongly support the view that FA+AA could inhibit MMP9 to exert a protective effect in cochlear hair cells during Cis injury.

The p38 MAPK pathway is involved in a variety of cellular processes, including immune response, cell survival, differentiation, and other signaling pathways [49]. Inhibiting p38 MAPK phosphorylation reduces the apoptotic pathway activation produced by hydrogen peroxide or Aβ1-42 [50]. Although several signaling pathways showed significant enrichment in our analysis, we focused on the MAPK signaling pathway due to its well-documented role in mediating cellular responses to oxidative stress, and apoptosis key mechanisms underlying Cis-induced toxicity. Furthermore, the MAPK pathway, particularly p38, has been implicated in regulating MMP9 expression, which aligns with our findings. Previous studies have shown that inhibiting MMP9 can protect hair cells and alleviate cochlear lateral wall damage caused by endotoxin injection into the tympanic cavity [51,52]. We found that FA+AA reduced p38 phosphorylation in HEI-OC1 cells and the cultured cochlear OHCs, and the p38MAPK inhibitor lowered the expression of p38 and MMP9 proteins, indicating that FA+AA could create a protective effect by inhibiting the p38 MAPK pathway and MMP9. The additive effects of FA and AA may be attributed to their complementary mechanisms of action in scavenging ROS and modulating apoptotic pathways. The upregulation of MMP9 has been implicated in Cis-induced tissue damage, particularly in the cochlea. Our findings suggest that FA and AA may exert their protective effects by inhibiting p38 MAPK activation and subsequent MMP9 upregulation. However, we cannot exclude the possibility that FA+AA may prevent oxidative damage by regulating other signaling pathways without further exploration. While we focused on p38 MAPK due to its established role in Cis-mediated oxidative stress and hair cell death, other MAPK branches—particularly JNK and ERK—are also critical regulators of cellular stress responses and survival. Our current data would not exclude the involvement of JNK or ERK, which could explain residual apoptosis in FA+AA-treated groups.

Our study focused on the fact that FA+AA has a better antioxidant function, and a reduction in the number of hair cells lost was observed through in vivo and in vitro experiments. Our data indicate a synergistic interaction between FA and AA in antioxidant activity; however, the absence of validated quantitative synergy parameters precludes the definitive characterization of this cooperative effect. Also, we only observed the effect on OHCs in these experiments. In addition to OHCs, Cis also impairs inner hair cells, spiral ganglion neurons, and stria vascularis. In future studies, it should be further clarified whether our treatment is equally effective in inner hair cells as well as in neurons connected to hair cells.

While our data demonstrated that FA+AA protect against Cis-induced ototoxicity, we emphasize the critical need to preserve the chemotherapeutic’s tumoricidal activity. Notably, sodium thiosulfate (STS)—currently the only FDA-approved otoprotectant (particularly for pediatric cases)—exemplifies the delicate risk–benefit balance in clinical practice. STS requires multiple administrations starting 6 h post Cis infusion, as improper timing may diminish Cis’s cytotoxicity and compromise oncological outcomes. Previous studies found that AA exhibits concentration-dependent effects: low doses may protect normal cells, while high doses can be pro-oxidant in tumors [53]. FA demonstrates a pro-oxidation effect at lower concentrations and an antioxidant effect at higher concentrations, promoting chemical resistance [54]. Combination therapy faces challenges in defining optimal FA and AA ratios to avoid interfering with Cis’s antitumor effects and mitigate potential pro-oxidant toxicities. Furthermore, the local application of FA+AA into the middle ear cavity presents a clinically relevant alternative to systemic delivery. The round window membrane permits the direct diffusion of drugs into the perilymph by passing the blood–labyrinth barrier. This achieves higher intracochlear drug concentrations with lower systemic doses, reducing off-target effects. Therefore, the successful clinical translation of FA+AA demands the precise optimization of both dosage parameters and delivery protocols, particularly regarding temporal separation from Cis infusion and localized administration routes.

Our study provides a thorough investigation of the effects of Cis, FA, and AA on cell viability, oxidative stress, and apoptosis, offering valuable insights into their mechanisms of action. By exploring the potential combined effects of FA and AA with Cis, we highlight novel therapeutic strategies that could mitigate Cis-induced toxicity while enhancing its efficacy. The findings have potential translational significance, particularly in improving the therapeutic index of Cis in cancer treatment. While our in vitro results are promising, further in vivo studies are needed to confirm the efficacy and safety of the combination treatment.

## 5. Conclusions

In conclusion, the current study demonstrates that combining FA with AA enhances the percentage of cochlear hair cell survival and improves hearing function following Cis administration. Our findings indicate that treatment with FA combined with AA lowers ROS generation and prevents apoptosis. This effect is mediated by inhibiting the expression of MMP9 and p38 in the p38/MAPK signaling pathway. Our data suggest that FA paired with AA may be an appealing therapeutic strategy in the clinical setting.

## Data Availability

The data presented in this study are available on request from the corresponding author. Original immunoblots are provided in the Supplementary Information file.

## Supplementary Materials

The following supporting information can be downloaded at: https://www.mdpi.com/article/10.3390/antiox14060619/s1, Figure S1: As illustrated in Figure 1A,B, immunofluorescence staining of cochlear outer hair cells revealed a notable decline in the number of these cells in mice subjected to cisplatin treatment, when compared to the control group. The damaged cells were highlighted using asterisks. ** *p* < 0.01; Figure S2: (A) Infrared spectral curves for the detection of substances in the solid state. The red curve represents the concentration of AA, while the black curve represents the concentration of FA. (B) Infrared spectral curves were observed in the liquid state. The red curve represents AA, the black curve represents FA, and the blue curve represents the two substances combined. The arrows are labeled near wavelengths of 500, 1000, and 1500 (cm^−1^). (C) CCK8 detects the effect of different concentrations of FA on HEI-OC1 cell viability. (D) CCK8 detects the effects of various AA concentrations on HEI-OC1 cell survival. (E,F) Hoechst staining was performed at both 0 h and 24 h across the four experimental groups: Con, Cis, FA, AA, and FA+AA. n = 6. Scale bars = 200 μm. (G) HEI-OC1 cells were preincubated with FA, AA, or a combination of FA and AA for 6 h, followed by stimulation with Cis for 24 h to assess cell viability. n = 6. Results are expressed as mean ± SEM. ** *p* < 0.01, *** *p* < 0.001; ## *p* < 0.01, ### *p* < 0.001; Figure S3: Hydrogen bond display during the docking of MMP9 with FA and AA; Figure S4: The objective of this study is to investigate the survival of extracochlear hair cells with topical application in the middle ear. (A) Immunofluorescence shows cochlear outer hair cells in each group. Scale bars = 25 μm. n = 3. (B) Statistical data showing cochlear outer hair cell survival in each group. (C) Immunofluorescence shows the expression of MMP9 in cochlear base, middle, and apex hair cells under the application of FA+AA. Scale bars = 10 μm. n = 6. Results are expressed as mean ± SEM. * *p* < 0.05, ** *p* < 0.01; ## *p* < 0.01, ### *p* < 0.001.

## Abbreviations

FA, ferulic acid; AA, ascorbic acid; Cis, cisplatin; ABR, auditory brainstem response; MMP9, matrix metalloproteinase 9; MAPK, mitogen-activated protein kinase; OHCs, outer hair cells; ROS, reactive oxygen species; TUNEL, terminal deoxynucleotidyl transferase-mediated dUTP nick-end-labeling; Bax, BCL 2-associated X; Bcl-2, B-cell lymphoma-2; CCK-8, Cell Counting Kit-8; Con, control; CETSA, cellular thermal shift assay; DARTS, drug affinity responsive target stability; NAC, N-acetyl-L-cysteine; HEI-OC1, House Ear Institute-Organ of Corti 1, a cochlear cell line that is used as an in vitro system for screening drug ototoxicity.

## References

[1] Dillard L.K., Lopez-Perez L., Martinez R.X., Fullerton A.M., Chadha S., McMahon C.M. (2022). Global burden of ototoxic hearing loss associated with platinum-based cancer treatment: A systematic review and meta-analysis. Cancer Epidemiol..

[2] Tang Q., Wang X., Jin H., Mi Y., Liu L., Dong M., Chen Y., Zou Z. (2021). Cisplatin-induced ototoxicity: Updates on molecular mechanisms and otoprotective strategies. Eur. J. Pharm. Biopharm..

[3] Yimit A., Adebali O., Sancar A., Jiang Y. (2019). Differential damage and repair of DNA-adducts induced by anti-cancer drug cisplatin across mouse organs. Nat. Commun..

[4] Mohan S., Smyth B.J., Namin A., Phillips G., Gratton M.A. (2014). Targeted amelioration of cisplatin-induced ototoxicity in guinea pigs. Otolaryngol.-Head Neck Surg..

[5] Borse V., Al Aameri R.F.H., Sheehan K., Sheth S., Kaur T., Mukherjea D., Tupal S., Lowy M., Ghosh S., Dhukhwa A. (2017). Epigallocatechin-3-gallate, a prototypic chemopreventative agent for protection against cisplatin-based ototoxicity. Cell Death Dis..

[6] Guo B., Chen J.H., Zhang J.H., Fang Y., Liu X.J., Zhang J., Zhu H.Q., Zhan L. (2023). Pattern-recognition receptors in endometriosis: A narrative review. Front. Immunol..

[7] Blaser H., Dostert C., Mak T.W., Brenner D. (2016). TNF and ROS Crosstalk in Inflammation. Trends Cell Biol..

[8] Kunnumakkara A.B., Thakur K.K., Rana V., Bora B., Banik K., Khatoon E., Sailo B.L., Shabnam B., Girisa S., Gupta S.C. (2019). Upside and Downside of Tumor Necrosis Factor Blockers for Treatment of Immune/Inflammatory Diseases. Crit. Rev. Immunol..

[9] Koo D.Y., Lee S.H., Lee S., Chang J., Jung H.H., Im G.J. (2016). Comparison of the effects of lipoic acid and glutathione against cisplatin-induced ototoxicity in auditory cells. Int. J. Pediatr. Otorhinolaryngol..

[10] Orgel E., Knight K.R., Chi Y.Y., Malvar J., Rushing T., Mena V., Eisenberg L.S., Rassekh S.R., Ross C.J.D., Scott E.N. (2023). Intravenous N-Acetylcysteine to Prevent Cisplatin-Induced Hearing Loss in Children: A Nonrandomized Controlled Phase I Trial. Clin. Cancer Res..

[11] Brock P.R., Maibach R., Childs M., Rajput K., Roebuck D., Sullivan M.J., Laithier V., Ronghe M., Dall’Igna P., Hiyama E. (2018). Sodium Thiosulfate for Protection from Cisplatin-Induced Hearing Loss. N. Engl. J. Med..

[12] Cabral-Pacheco G.A., Garza-Veloz I., Castruita-De la Rosa C., Ramirez-Acuña J.M., Perez-Romero B.A., Guerrero-Rodriguez J.F., Martinez-Avila N., Martinez-Fierro M.L. (2020). The Roles of Matrix Metalloproteinases and Their Inhibitors in Human Diseases. Int. J. Mol. Sci..

[13] Cheung S.W., Bhavnani E., Simmons D.G., Bellingham M.C., Noakes P.G. (2024). Perineuronal nets are phagocytosed by MMP-9 expressing microglia and astrocytes in the SOD1^G93A^ ALS mouse model. Neuropathol. Appl. Neurobiol..

[14] Li S., Zheng L., Zhang J., Liu X., Wu Z. (2021). Inhibition of ferroptosis by up-regulating Nrf2 delayed the progression of diabetic nephropathy. Free Radic. Biol. Med..

[15] Trotta M.C., Pieretti G., Petrillo F., Alessio N., Hermenean A., Maisto R., D’Amico M. (2020). Resolvin D1 reduces mitochondrial damage to photoreceptors of primary retinal cells exposed to high glucose. J. Cell. Physiol..

[16] Setz C., Brand Y., Radojevic V., Hanusek C., Mullen P.J., Levano S., Listyo A., Bodmer D. (2011). Matrix metalloproteinases 2 and 9 in the cochlea: Expression and activity after aminoglycoside exposition. Neuroscience.

[17] Sung M., Wei E., Chavez E., Jain N., Levano S., Binkert L., Ramseier A., Setz C., Bodmer D., Ryan A.F. (2014). Inhibition of MMP-2 but not MMP-9 influences inner ear spiral ganglion neurons in vitro. Cell. Mol. Neurobiol..

[18] Wu J., Han W., Chen X., Guo W., Liu K., Wang R., Zhang J., Sai N. (2017). Matrix metalloproteinase-2 and -9 contribute to functional integrity and noise-induced damage to the blood-labyrinth-barrier. Mol. Med. Rep..

[19] Hu B.H., Cai Q., Hu Z., Patel M., Bard J., Jamison J., Coling D. (2012). Metalloproteinases and their associated genes contribute to the functional integrity and noise-induced damage in the cochlear sensory epithelium. J. Neurosci..

[20] Matusiak M., Oziębło D., Ołdak M., Rejmak E., Kaczmarek L., Skarżyński P.H., Skarżyński H. (2022). Prospective cohort study reveals MMP-9, a neuroplasticity regulator, as a prediction marker of cochlear implantation outcome in prelingual deafness treatment. Mol. Neurobiol..

[21] Yu M., Jiang W.J., Yu M., Zhou Z., Wang M., Li L. (2025). The Upregulation of IL-1β Induced by Cisplatin Triggers PI3K/AKT/MMP9 Pathway in Pericytes Mediating the Leakage of the Blood Labyrinth Barrier. J. Inflamm. Res..

[22] Luo L., Yang J.X., Luo T., Liu D., Wu G.H., He J.M. (2021). A study on the mechanism of PP2A in the recovery of SCI in rats through downregulation of MMP-9 via MAPK signaling pathway. Eur. Rev. Med. Pharmacol. Sci..

[23] Huang Q., Lan F., Wang X., Yu Y., Ouyang X., Zheng F., Han J., Lin Y., Xie Y., Xie F. (2014). IL-1β-induced activation of p38 promotes metastasis in gastric adenocarcinoma via upregulation of AP-1/c-fos, MMP2 and MMP9. Mol. Cancer.

[24] Jo E.R., Youn C.K., Jun Y., Cho S.I. (2019). The protective role of ferulic acid against cisplatin-induced ototoxicity. Int. J. Pediatr. Otorhinolaryngol..

[25] Zadrozniak M., Szymanski M., Luszczki J.J. (2019). Vitamin C alleviates ototoxic effect caused by coadministration of amikacin and furosemide. Pharmacol. Rep..

[26] Yogeeta S.K., Gnanapragasam A., Kumar S.S., Subhashini R., Sathivel A., Devaki T. (2006). Synergistic interactions of ferulic acid with ascorbic acid: Its cardioprotective role during isoproterenol induced myocardial infarction in rats. Mol. Cell. Biochem..

[27] Yogeeta S.K., Raghavendran H.R., Gnanapragasam A., Subhashini R., Devaki T. (2006). Ferulic acid with ascorbic acid synergistically extenuates the mitochondrial dysfunction during beta-adrenergic catecholamine induced cardiotoxicity in rats. Chem.-Biol. Interact..

[28] Lin F.H., Lin J.Y., Gupta R.D., Tournas J.A., Burch J.A., Selim M.A., Monteiro-Riviere N.A., Grichnik J.M., Zielinski J., Pinnell S.R. (2005). Ferulic acid stabilizes a solution of vitamins C and E and doubles its photoprotection of skin. J. Investig. Dermatol..

[29] Teitz T., Fang J., Goktug A.N., Bonga J.D., Diao S., Hazlitt R.A., Iconaru L., Morfouace M., Currier D., Zhou Y. (2018). CDK2 inhibitors as candidate therapeutics for cisplatin- and noise-induced hearing loss. J. Exp. Med..

[30] He Y., Li W., Zheng Z., Zhao L., Li W., Wang Y., Li H. (2020). Inhibition of Protein arginine methyltransferase 6 reduces reactive oxygen species production and attenuates aminoglycoside- and cisplatin-induced hair cell death. Theranostics.

[31] He Y., Zheng Z., Liu C., Li W., Zhao L., Nie G., Li H. (2022). Inhibiting DNA methylation alleviates cisplatin-induced hearing loss by decreasing oxidative stress-induced mitochondria-dependent apoptosis via the LRP1-PI3K/AKT pathway. Acta Pharm. Sin. B.

[32] Liu W., Xu L., Wang X., Zhang D., Sun G., Wang M., Wang M., Han Y., Chai R., Wang H. (2021). PRDX1 activates autophagy via the PTEN-AKT signaling pathway to protect against cisplatin-induced spiral ganglion neuron damage. Autophagy.

[33] Martinez Molina D., Jafari R., Ignatushchenko M., Seki T., Larsson E.A., Dan C., Sreekumar L., Cao Y., Nordlund P. (2013). Monitoring drug target engagement in cells and tissues using the cellular thermal shift assay. Science.

[34] Yang Z., Zhang X.W., Zhuo F.F., Liu T.T., Luo Q.W., Zheng Y.Z., Li L., Yang H., Zhang Y.C., Wang Y.H. (2023). Allosteric Activation of Transglutaminase 2 via Inducing an “Open” Conformation for Osteoblast Differentiation. Adv. Sci..

[35] Lomenick B., Jung G., Wohlschlegel J.A., Huang J. (2011). Target identification using drug affinity responsive target stability (DARTS). Curr. Protoc. Chem. Biol..

[36] Chattaraj A., Syed M.P., Low C.A., Owonikoko T.K. (2023). Cisplatin-Induced Ototoxicity: A Concise Review of the Burden, Prevention, and Interception Strategies. JCO Oncol. Pract..

[37] Santos N.A.G.D., Ferreira R.S., Santos A.C.D. (2020). Overview of cisplatin-induced neurotoxicity and ototoxicity, and the protective agents. Food Chem. Toxicol. Int. J. Publ. Br. Ind. Biol. Res. Assoc..

[38] Yu D., Gu J., Chen Y., Kang W., Wang X., Wu H. (2020). Current Strategies to Combat Cisplatin-Induced Ototoxicity. Front. Pharmacol..

[39] Freyer D.R., Brock P.R., Chang K.W., Dupuis L.L., Epelman S., Knight K., Mills D., Phillips R., Potter E., Risby D. (2020). Prevention of cisplatin-induced ototoxicity in children and adolescents with cancer: A clinical practice guideline. Lancet Child Adolesc. Health.

[40] Zduńska K., Dana A., Kolodziejczak A., Rotsztejn H. (2018). Antioxidant Properties of Ferulic Acid and Its Possible Application. Ski. Pharmacol. Physiol..

[41] Celebi S., Gurdal M.M., Ozkul M.H., Yasar H., Balikci H.H. (2013). The effect of intratympanic vitamin C administration on cisplatin-induced ototoxicity. Eur. Arch. Oto-Rhino-Laryngol..

[42] Gong L., Chen B., Chen J., Li Y. (2022). Protective Effects of Vitamin C against Neomycin-Induced Apoptosis in HEI-OC1 Auditory Cell. Neural Plast..

[43] Yogeeta S.K., Gnanapragasam A., Senthilkumar S., Subhashini R., Devaki T. (2006). Synergistic salubrious effect of ferulic acid and ascorbic acid on membrane-bound phosphatases and lysosomal hydrolases during experimental myocardial infarction in rats. Life Sci..

[44] Sheth S., Mukherjea D., Rybak L.P., Ramkumar V. (2017). Mechanisms of Cisplatin-Induced Ototoxicity and Otoprotection. Front. Cell. Neurosci..

[45] Schacht J., Talaska A.E., Rybak L.P. (2012). Cisplatin and aminoglycoside antibiotics: Hearing loss and its prevention. Anat. Rec..

[46] Kros C.J., Steyger P.S. (2019). Aminoglycoside- and Cisplatin-Induced Ototoxicity: Mechanisms and Otoprotective Strategies. Cold Spring Harb. Perspect. Med..

[47] Wei S., Niu M., Wang J., Wang J., Su H., Luo S., Zhang X., Guo Y., Liu L., Liu F. (2016). A network pharmacology approach to discover active compounds and action mechanisms of San-Cao Granule for treatment of liver fibrosis. Drug Des. Dev. Ther..

[48] Yadav S.K., Kambis T.N., Kar S., Park S.Y., Mishra P.K. (2020). MMP9 mediates acute hyperglycemia-induced human cardiac stem cell death by upregulating apoptosis and pyroptosis in vitro. Cell Death Dis..

[49] Canovas B., Nebreda A.R. (2021). Diversity and versatility of p38 kinase signalling in health and disease. Nat. Rev. Mol. Cell Biol..

[50] Chen K., Lu Y., Liu C., Zhang L., Fang Z., Yu G. (2018). Morroniside prevents H_2_O_2_ or Aβ_1-42_-induced apoptosis via attenuating JNK and p38 MAPK phosphorylation. Eur. J. Pharmacol..

[51] Jang C.H., Cho Y.B., Choi C.H., Jang Y.S., Jang S.J., Jung W.K., Park B.Y., Kim M.Y. (2014). ALH-L1005 attenuates endotoxin induced inner ear damage. Int. J. Pediatr. Otorhinolaryngol..

[52] Choi C.H., Jang C.H., Cho Y.B., Jo S.Y., Kim M.Y., Park B.Y. (2012). Matrix metalloproteinase inhibitor attenuates cochlear lateral wall damage induced by intratympanic instillation of endotoxin. Int. J. Pediatr. Otorhinolaryngol..

[53] Ma Y., Chapman J., Levine M., Polireddy K., Drisko J., Chen Q. (2014). High-dose parenteral ascorbate enhanced chemosensitivity of ovarian cancer and reduced toxicity of chemotherapy. Sci. Transl. Med..

[54] Paciello F., Fetoni A.R., Mezzogori D., Rolesi R., Di Pino A., Paludetti G., Grassi C., Troiani D. (2020). The dual role of curcumin and ferulic acid in counteracting chemoresistance and cisplatin-induced ototoxicity. Sci. Rep..

