# Supplementary material for: Ferulic Acid Combines with Ascorbic Acid to Target MMP9 to Attenuate Cisplatin-Induced Ototoxicity Through the p38MAPK Signaling Pathway
Source: Antioxidants (Basel). 2025 May 22;14(6):619. doi: 10.3390/antiox14060619 (PMC12189926; doi:10.3390/antiox14060619)
Supplement: Supplementary file 1 [file antioxidants-14-00619-s001.zip › antioxidants-3604301-supplementary.pdf]

# Figure S1

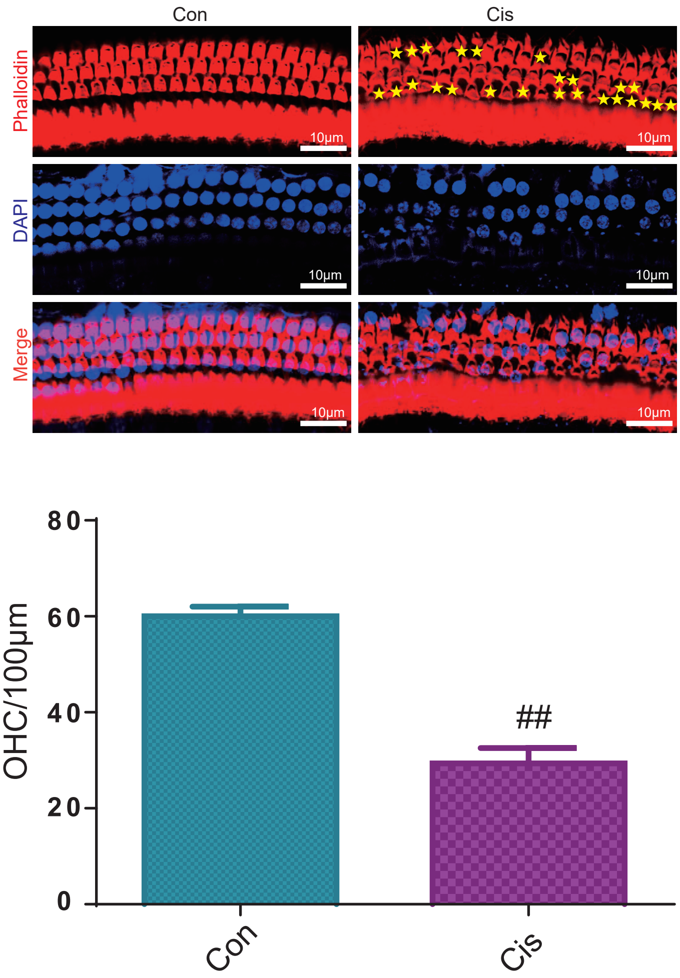
Con
Cis
Phalloidin
DAPI
Merge
10μm
OHC/100μm
80
60
40
20
0
##
Con
Cis

Figure S2

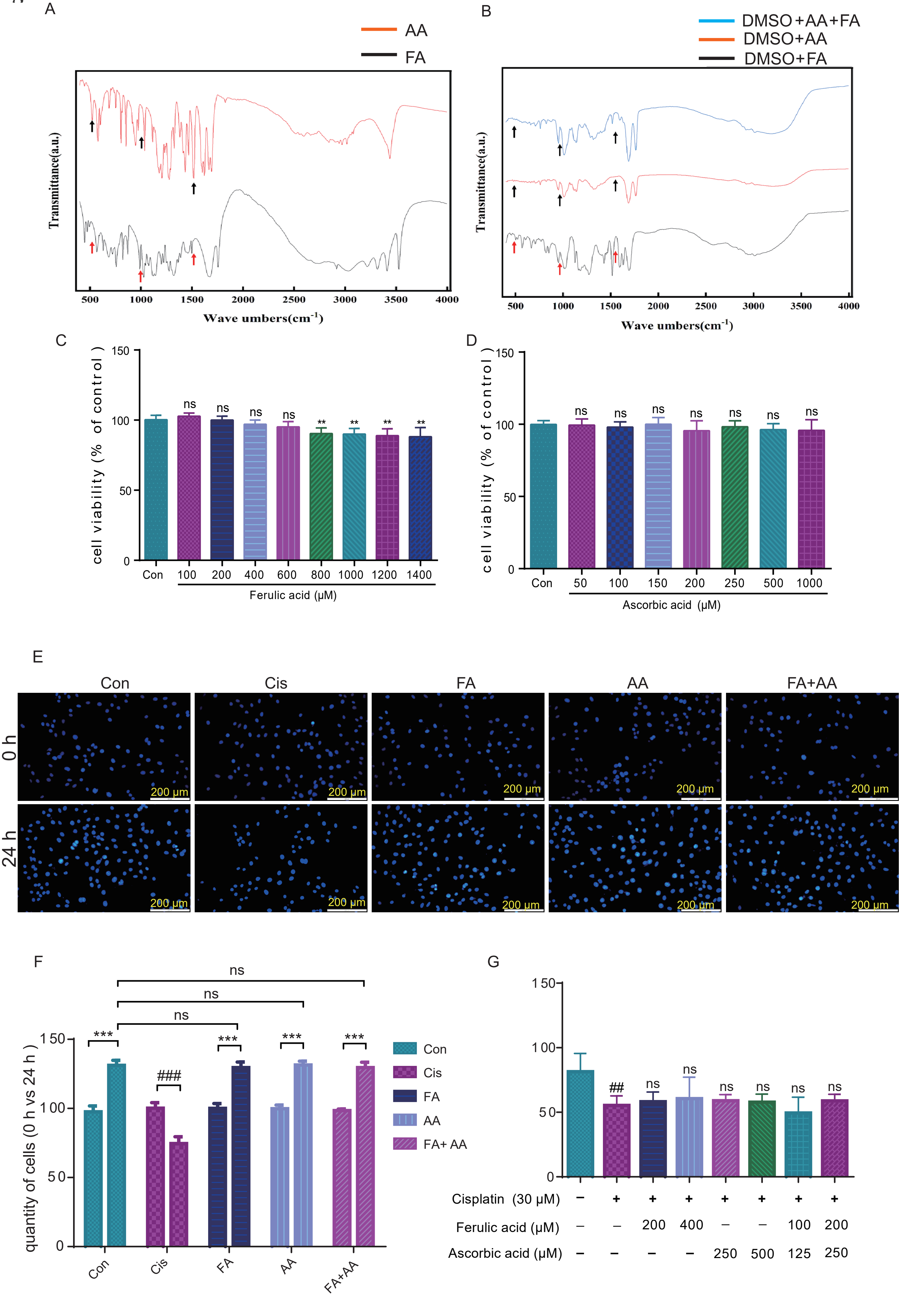
A
AA
FA
Transmittance(a.u.)
Wave umbers(cm$^{-1}$)
500 1000 1500 2000 2500 3000 3500 4000
B
DMSO+AA+FA
DMSO+AA
DMSO+FA
Transmittance(a.u.)
Wave umbers(cm$^{-1}$)
500 1000 1500 2000 2500 3000 3500 4000
C
cell viability (% of control)
150 100 50 0
Con 100 200 400 600 800 1000 1200 1400
Ferulic acid (μM)
ns ns ns ns ** ** ** **
D
cell viability (% of control)
150 100 50 0
Con 50 100 150 200 250 500 1000
Ascorbic acid (μM)
ns ns ns ns ns ns ns
E
Con
Cis
FA
AA
FA+AA
0 h
24 h
200 μm
F
quantity of cells (0 h vs 24 h)
150 100 50 0
Con Cis FA AA FA+AA
*** ### *** *** ***
ns ns ns
Con
Cis
FA
AA
FA+ AA
G
150 100 50 0
## ns ns ns ns ns ns
Cisplatin (30 μM) – + + + + + + +
Ferulic acid (μM) – – 200 400 – – 100 200
Ascorbic acid (μM) – – – – 250 500 125 250

# Figure S3

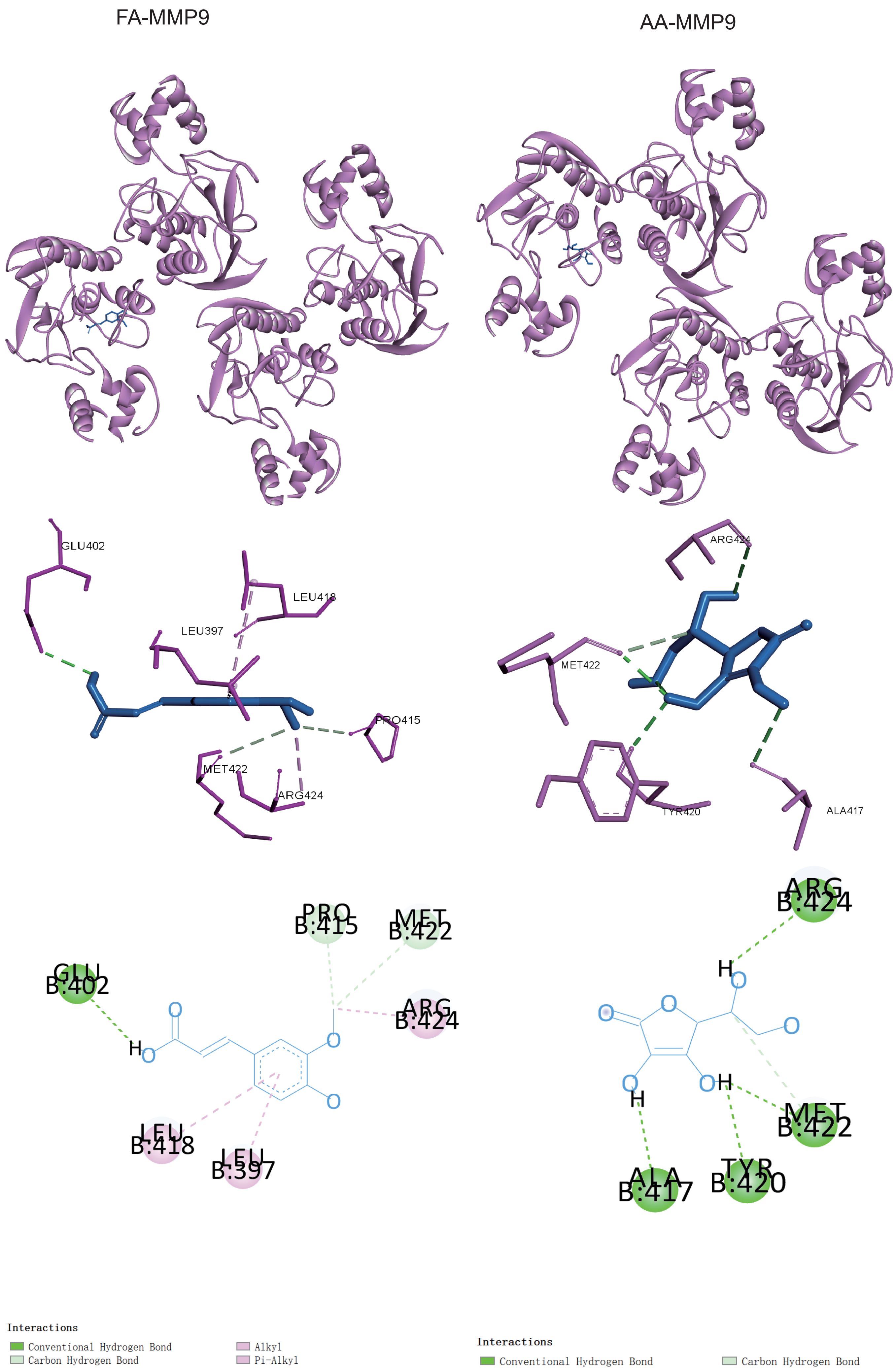
FA-MMP9
AA-MMP9
GLU402
LEU418
LEU397
PRO415
MET422
ARG424
ARG424
MET422
TYR420
ALA417
PRO B:415
MET B:422
GLU B:402
ARG B:424
LEU B:418
LEU B:397
ARG B:424
MET B:422
ALA B:417
TYR B:420
Interactions
Conventional Hydrogen Bond
Carbon Hydrogen Bond
Alkyl
Pi-Alkyl
Interactions
Conventional Hydrogen Bond
Carbon Hydrogen Bond

# Figure S4

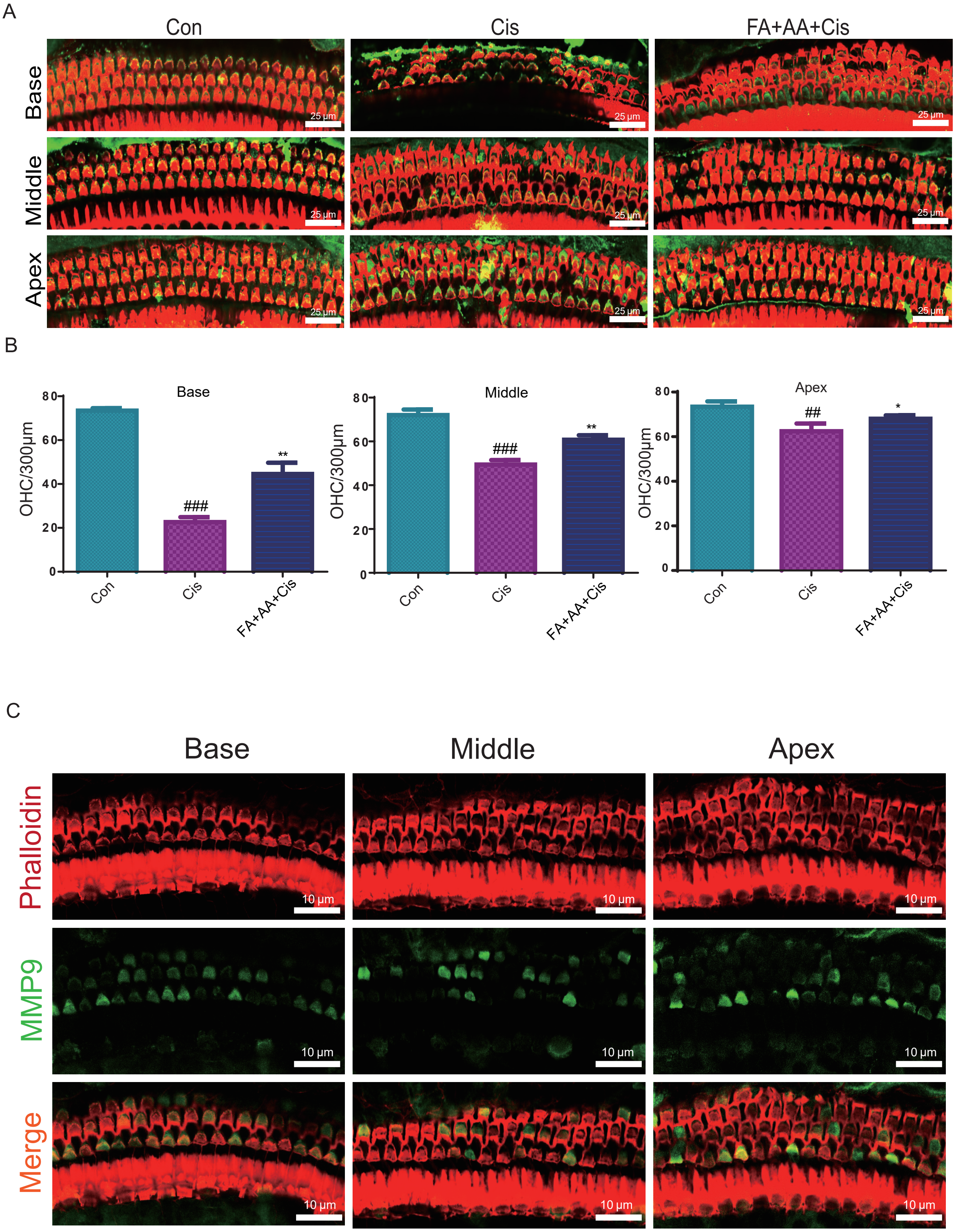
A
Con
Cis
FA+AA+Cis
Base
Middle
Apex
25 μm
B
Base
Middle
Apex
OHC/300μm
0
20
40
60
80
###
**
##
*
Con
Cis
FA+AA+Cis
C
Base
Middle
Apex
Phalloidin
MMP9
Merge
10 μm
